# Injectable Hydrogels for Breast Cancer Therapy: From Tumor Microenvironment-Responsive and Actively Targeted Drug Delivery to Immunotherapy and Theranostics

**DOI:** 10.3390/pharmaceutics18080979

**Published:** 2026-08-09

**Authors:** Yuhang Jiao, Huiling Zuo, Jiaxin Chen, Shihao Zheng, Sen Tong, Xiaoyi Feng, Wei Zhao

**Affiliations:** 1 Yunnan Key Laboratory of Integrated Traditional Chinese and Western Medicine for Chronic Disease in Prevention and Treatment, Yunnan University of Chinese Medicine, Kunming 650500, China; 2 Xiyuan Hospital, China Academy of Chinese Medical Sciences, Beijing 100091, China

**Keywords:** injectable hydrogels, controlled-release drug delivery systems, tumor microenvironment, ligand modification, breast cancer

## Abstract

Breast cancer treatment still faces challenges including local recurrence, systemic toxicity, tumor heterogeneity, drug resistance, and immunosuppression. Conventional systemic administration provides limited exposure at the tumor site and exhibits significant toxicity. Injectable hydrogels, combining the properties of minimally invasive administration, in situ gelation, local retention, and sustained release, have become a key platform for local precision drug delivery. Compared with nanomedicines or free drugs, hydrogels can both prolong drug retention time and achieve on-demand release through the modulation of crosslinking density, degradation rate, and responsive chemical bonds. This review is organized around the material logic of such systems. Injectable hydrogels are first classified into natural, synthetic, hybrid, supramolecular, nanocomposite, and self-healing systems, the in situ gelation chemistries available to each are compared, and network parameters such as crosslinking density, mesh size, swelling, porosity, modulus, and rheology are related to release kinetics and intratumoral retention. Current research is primarily advancing along two directions: one is the construction of pH-, enzyme-, redox/ROS-, hypoxia-, ATP-, glucose-or thermo-responsive hydrogels; the other is achieving active targeting by integrating functionalized hydrogels with targets such as CD44, folate receptor, integrins, EGFR, transferrin receptor, and HER2 or with biomimetic cell-membrane coatings. On this basis, hydrogels have been extended to cancer vaccines, immune checkpoint modulation, local delivery of CAR-T/CAR-NK, as well as combination therapies involving chemotherapy, photothermal therapy, photodynamic therapy, chemodynamic therapy, sonodynamic therapy, radiosensitization, gene therapy, and theranostics. The constraints imposed on hydrogel design by different payload classes, including small molecules, natural products, proteins and peptides, nucleic acids, antibodies, exosomes, and gene-editing machinery, are further examined, and imaging-integrated theranostic gels are discussed together with the emerging role of machine learning and digital fabrication in hydrogel optimization. Based on the biological foundations of breast cancer, this review summarizes advances in the material design, microenvironment-responsive release, targeting strategies, immunomodulation, and combination therapy of hydrogels, critically evaluates the limitations of each strategy, and aims to provide a reference for the design of mechanistically well-defined and translatable hydrogel delivery systems for breast cancer.

## 1. Introduction

Among women worldwide, breast cancer ranks as one of the most frequently encountered invasive malignancies and constitutes a major hazard to female health [1]. By 2024, this disease made up roughly 32% of all cancers diagnosed in women, while its associated deaths accounted for about 15% of the total [2]. Data most recently published by the American Cancer Society indicate that breast cancer cases kept climbing throughout 2024, and the disease continues to be the second most frequent cause of cancer-related mortality among women [3]. Even though both diagnostic methods and treatment strategies have advanced steadily over the past few years, the elevated rates of occurrence and relapse associated with this malignancy have not declined. Currently, clinical management still depends largely on traditional approaches including surgical excision, chemotherapy, and radiotherapy, each carrying its own drawbacks. Surgical resection cannot completely eliminate the cancer cells infiltrating the tissue surrounding the tumor, resulting in a markedly increased rate of postoperative recurrence [4]. In addition, chemotherapy is administered systemically and therefore lacks selectivity between tumor tissue and normal tissue; while killing tumor cells, it also damages normal tissue, causing adverse effects, and long-term use leads to drug resistance. Similarly, radiotherapy induces fibrosis around the tumor tissue, damages blood vessels, and suppresses the immune system, thereby affecting patient prognosis [5]. To overcome these difficulties, it is essential to develop a novel drug delivery system that precisely targets the local tumor tissue, so as to improve drug bioavailability, reduce damage to normal tissue, and lower systemic cytotoxicity.

Over the past several years, scientists have engineered numerous precise, target-specific controlled-release platforms aimed at the localized management of breast cancer, capitalizing on the injectability, in situ gel formation, and good biocompatibility that hydrogels offer. Within this category, injectable hydrogel systems that respond to the tumor microenvironment have emerged by leveraging the pathological traits unique to the breast cancer tumor microenvironment [6]. The principal characteristics of the breast cancer tumor microenvironment include an acidic microenvironment with a pH of 6.5–6.9, a redox microenvironment with elevated GSH, a hyperthermic microenvironment with increased temperature, and an enzymatic microenvironment with overexpression of specific enzymes [7]. Based on these microenvironmental stimuli, pH-sensitive groups, disulfide bonds, thermosensitive materials, or enzyme-responsive peptide segments are incorporated into hydrogels, enabling them to respond specifically to the corresponding tumor microenvironment and achieve a precise controlled drug-release mode. In addition, active ligand–modified hydrogels exert active targeted drug delivery through ligand–receptor binding [8]. Breast cancer tumor cells overexpress certain specific receptors on their surface, such as the CD44 receptor, the folate receptor, and certain specific antigens [9]. Therefore, by designing hydrogels whose surfaces are modified with corresponding ligands (such as small molecules, peptides, and aptamers) or that encapsulate ligand-modified nanocarriers, active recognition of and specific binding to breast cancer cells can be achieved, significantly enhancing cellular drug uptake. Ligand modification not only improves the tumor-targeting enrichment capacity of the hydrogel delivery system but also exploits receptor-mediated endocytosis to promote drug entry into cells, thereby enhancing antitumor capacity.

This review analyzes the advantages of tumor microenvironment-responsive hydrogels and active ligand-modified hydrogels in targeted breast cancer delivery, laying the groundwork for the future development of more precise hydrogel delivery platforms. It further discusses the applications of such local delivery systems in breast cancer diagnosis and in various therapeutic approaches (such as immunotherapy and combination therapy), with the aim of providing a theoretical basis for the development of multimodal breast cancer treatment platforms.

## 2. Biological Basis for Precise Delivery in Breast Cancer: Tumor Microenvironment and Active Targets

Serving as an intricate ecosystem upon which malignant cells rely for their viability and advancement, the tumor microenvironment (TME) is deeply involved in driving the onset and progression of breast cancer [10]. In contrast to healthy tissue, the TME displays unique biochemical characteristics, including a lowered pH, heightened enzymatic activity, strong redox potential, and raised temperature. Lately, investigators have concentrated on harnessing these characteristics to construct drug delivery platforms directed at tumor tissue, seeking to enhance drug biocompatibility, bioavailability, targeting efficiency, and controlled release, while simultaneously reshaping the tumor microenvironment, slowing tumor advancement, and blocking immune evasion.

Within breast cancer lesions, the pH outside the cells falls to around 6.5–6.9, reflecting a pronounced acidic nature [11]. These distinctive physicochemical features of the tumor region are capable of inducing conformational alterations in hydrogels sensitive to pH, which in turn facilitates the liberation of drugs precisely where the tumor resides.

A hallmark of the redox-disrupted tumor region is the markedly raised level of intracellular glutathione (GSH) found within breast cancer cells [12]. Such a strongly reductive setting strengthens the capacity of malignant cells to withstand oxidative stress and to multiply [13,14]. Nevertheless, this abundant GSH likewise furnishes the foundation for engineering redox-responsive hydrogels [15]. When disulfide bonds are incorporated as crosslinking elements within the hydrogel framework, the abundant GSH present inside tumor cells is able to cleave these disulfide linkages, prompting swift breakdown of the hydrogel architecture along with drug release. Furthermore, this stimulus-responsive behavior not only secures effective drug delivery inside malignant cells but also guards against early leakage in the extracellular space and throughout blood circulation.

Beyond this, the tumor region is marked by heightened release of numerous enzymes, including hyaluronidase and matrix metalloproteinases, each possessing distinct chemical attributes [16]. Of these, matrix metalloproteinases (MMPs)—a group of zinc-reliant endopeptidases—show abnormally high expression throughout the breast cancer tumor microenvironment. Excessive levels of MMP-2 and MMP-9 are tightly linked to the invasive and metastatic behavior of breast cancer [17]. Through the integration of MMP-cleavable peptide motifs (for instance, GPLGVRGK and PVGLIG) as crosslinking junctions within the hydrogel network, MMP-driven degradation of the hydrogel can take place specifically where the tumor is located, thereby enabling accurate drug liberation [18]. Moreover, this enzyme-triggered approach may be paired with additional responsive strategies to build multi-responsive intelligent drug delivery platforms, thus further refining the accuracy of targeted drug delivery.

Three further metabolic features of the breast tumor deserve attention because they underpin the responsive chemistries. First, the disordered vasculature of the breast tumor leaves large regions at oxygen tensions well below those of normal mammary tissue, and the resulting stabilization of hypoxia-inducible factor 1α raises the activity of nitroreductases and azoreductases—the enzymes that reduce nitroimidazole and azobenzene motifs and thereby provide a chemical handle for hypoxia-triggered release [19]. Second, aerobic glycolysis raises glucose consumption and lactate output, so that phenylboronic acid–diol equilibria and glucose oxidase-based systems can be coupled to the tumor’s own metabolic flux [20]. Third, adenosine triphosphate accumulates in the tumor interstitium at concentrations orders of magnitude above those of healthy tissue, and remains in the low millimolar range in the cytosol, which is sufficient to displace aptamer or phenylboronate crosslinks in ATP-sensitive networks [21]. Each of these gradients is, however, considerably less reproducible between patients than the acidic and enzymatic signatures discussed above, and this variability is a recurring source of the inconsistent release behavior noted later in this review.

Moreover, apart from engineering responsive hydrogels grounded in the pathological and biochemical traits of the tumor region, the abnormally high abundance of certain receptors found on breast cancer cell membranes supplies target molecules that enable the creation of ligand-decorated targeting hydrogels [22]. CD44, which serves as the chief membrane receptor recognizing hyaluronic acid, becomes activated and strongly expressed across breast cancer cells, while it stays dormant on healthy cells [23]. For this reason, hyaluronic acid may be employed to functionalize drug carriers like hydrogels so as to home in on malignant cells while sparing healthy tissue from harm [24]. In a comparable manner, the folate receptor (FR) is present at elevated amounts across a range of cancerous cells, breast cancer among them, yet appears only sparingly in normal tissue [25]. Since folate constitutes a vital vitamin required for nucleotide synthesis amid cell division, fast-dividing tumor cells boost folate receptor levels to satisfy their intense metabolic needs [26]. Such a disparity in receptor abundance offers a basis for folate ligand–functionalized targeted drug carriers. Additionally, human epidermal growth factor receptor 2 (HER2) shows amplification or excessive expression in roughly 20–25% of breast cancer cases and correlates with tumor aggressiveness and unfavorable outcomes [27]. Being a firmly established clinical target, HER2 not only represents a key therapeutic objective but also offers a targeting foundation for antibody-modified drug delivery platforms [28].

The receptor repertoire available for ligand design extends well beyond these three. Integrins of the αvβ3 and α5β1 families are upregulated on tumor neovasculature and on invasive breast cancer cells, and are addressed by the RGD motif and its cyclic and tumor-penetrating derivatives; epidermal growth factor receptor (EGFR, also designated HER1) is overexpressed in a substantial fraction of triple-negative disease, for which no hormonal or HER2-directed option exists; and the transferrin receptor (CD71) is upregulated in proliferating tumor cells to sustain iron uptake and has long served as a portal for receptor-mediated endocytosis [29,30]. A complementary strategy dispenses with a defined ligand altogether and coats the carrier with a cell membrane—erythrocyte, platelet, leukocyte, or homotypic tumor cell membrane—so that the native surface proteome performs the recognition [31,32].

Notably, these unique pathological and biochemical features of the tumor microenvironment, together with the differential expression of receptors on the tumor cell surface, collectively constitute the theoretical basis for the design of intelligent responsive hydrogels and ligand-modified hydrogels, providing molecular targets for achieving precise treatment of breast cancer.

## 3. Material Design of Injectable Hydrogels for Precise Delivery

The core of using injectable hydrogels for precise breast cancer delivery is not merely loading drugs into a gel. Rather, through the coordinated design of material structure, gelation mechanism, degradation behavior, and cargo release mode, the hydrogel is transformed into a therapeutic platform that can be locally injected, retained in situ, and controllably released. Accordingly, material design should center on four objectives: forming a stable drug depot within the tumor, the peritumoral region, or the postoperative tumor bed; achieving sustained or on-demand release of the therapeutic cargo; preserving the activity of drugs, proteins, nucleic acids, nanoparticles, or immune cells in the local microenvironment; and providing an engineerable foundation for the tumor microenvironment responsiveness, active targeting, and multimodal combination therapy discussed in the following sections.

### 3.1. Classification and Physicochemical Characteristics of Injectable Hydrogels

Injectable hydrogels for breast cancer delivery mainly comprise natural polymer hydrogels (e.g., hyaluronic acid, chitosan, sodium alginate, gelatin, collagen, and dextran), synthetic polymer hydrogels (e.g., PEG, PVA, PNIPAM, and PLGA-PEG-PLGA), self-assembling peptide or DNA hydrogels, hybrid hydrogels, nanocomposite hydrogels, and injectable self-healing systems [33,34]. Natural polymers offer favorable biocompatibility, biodegradability, and tissue affinity; in particular, hyaluronic acid can additionally target the CD44 receptor, and chitosan confers cationic, adhesive, and pH-sensitive properties [35]. Synthetic polymers provide a well-defined structure, good batch-to-batch consistency, and tunable mechanical and degradation behavior, but their limited bioactivity usually requires further functionalization with peptides, ligands, degradable bonds, or responsive groups. Peptide and DNA hydrogels enable the integration of enzyme-cleavable sites, targeting sequences, and nucleic acid drug delivery, whereas nanocomposite hydrogels embed liposomes, polymeric nanoparticles, metal–organic frameworks, two-dimensional or photothermal nanomaterials, or biomimetic vesicles to increase drug loading, enhance local retention, and confer photothermal, photodynamic, magnetothermal, imaging, or immunomodulatory functions [36,37].

These materials are grouped here by the origin of the matrix and by the mode of network assembly, since these two attributes set the range of physicochemical properties that a depot can attain. Hybrid and supramolecular hydrogels are treated as families in their own right: the former is adopted where neither a natural nor a synthetic component alone provides both drug-loading capacity and mechanical persistence, whereas the latter gels through reversible non-covalent interactions and shear-thins during injection, which is decisive for protein and cell cargo. Injectable self-healing systems, built from reversible bonds that reform after needle shear, constitute a functional rather than a compositional class and can be constructed from any of the matrices above. The modulus, swelling behaviour, degradation route, drug-loading capacity, and injectability of each family are summarised in Table 1.

### 3.2. In Situ Gelation Mechanisms

In situ gelation is the key determinant of the local application of injectable hydrogels. Common mechanisms include temperature-triggered gelation, ionic crosslinking, dynamic covalent crosslinking (Schiff base, hydrazone, boronated ester, and disulfide bonds), host–guest interactions, supramolecular self-assembly, and photo- or enzymatic crosslinking [50]. These mechanisms are compared below in terms of gelation rate, cargo compatibility, and network persistence.

The selection of a gelation chemistry is governed by three practical requirements. Gelation must be rapid enough to prevent the precursor solution from draining out of the injection track, yet slow enough to permit complete extrusion through a clinically applicable needle; the crosslinking reaction must not damage the loaded cargo; and the resulting network must remain intact for at least as long as the intended release period. Physical crosslinking mechanisms satisfy the second requirement most readily. Poloxamer 407 exists as dispersed micelles at low temperature, and these micelles pack into a three-dimensional network once the critical gelation temperature is reached. The sol–gel transition point can therefore be adjusted to approximately 37 °C by varying the polymer concentration or by grafting the copolymer onto a hydrophilic backbone, and no reactive species are produced during this process [51]. PLGA-PEG-PLGA triblock copolymers gel through the same hydrophobic mechanism and additionally undergo ester hydrolysis, so that the retention time of the depot is determined by the length of the polyester blocks [52]. Because no reactive chemistry is involved, most protein- and cell-loaded depots reported for breast cancer are based on thermosensitive systems. Ionic crosslinking is likewise mild, occurring through the egg-box association of guluronate blocks in alginate with Ca^2+^ and through the interaction of chitosan with beta-glycerophosphate; however, the resulting networks gradually soften as counterions exchange with the interstitial fluid [53]. In addition, host-guest inclusion, typically between beta-cyclodextrin and adamantane or azobenzene, and peptide self-assembly both produce shear-thinning gels. The viscosity of such gels decreases under the shear generated during injection and recovers within seconds afterwards, so that encapsulated cells are exposed to only low stress during extrusion [54].

Compared with physical mechanisms, covalent crosslinking provides better control over network persistence, but greater attention must be paid to cargo compatibility. Schiff base formation between aldehyde-bearing polysaccharides and the amino groups of chitosan, gelatin, or peptides proceeds within minutes at neutral pH and requires neither catalyst nor initiator. The resulting imine bond is dynamic: it undergoes continuous exchange, which confers self-healing after needle shear, and it hydrolyzes preferentially as the pH decreases, so that the crosslink itself serves as the release trigger, as in the CMCS/OHA-Met hydrogel [55]. Michael addition between thiols and acrylates or maleimides, together with the closely related thiol-ene coupling, is similarly rapid at physiological pH and is commonly adopted when a defined and essentially irreversible network is required. Because free thiols are consumed during the reaction, however, cysteine-containing proteins may be modified [56]. Strain-promoted azide-alkyne cycloaddition and tetrazine-trans-cyclooctene ligation proceed without catalyst, initiator, or illumination and do not react with functional groups native to tissue, and are therefore particularly suitable when the network must be assembled around sensitive biologics or living cells. Gelation times of approximately one to ten minutes can be obtained by varying the degree of substitution and the polymer concentration, although the cost of the functionalized precursors has so far limited their application [57]. Photo-crosslinking of methacrylated gelatin or hyaluronic acid affords stiff and finely tunable networks under spatial and temporal control and can now be initiated with visible light. Nevertheless, the radicals generated during initiation attack nucleic acids and membrane lipids, and the light source must reach the injection site, which restricts this approach to superficial lesions and to intraoperative use [58]. Enzymatic crosslinking operates under fully physiological conditions. Horseradish peroxidase couples tyramine- or phenol-conjugated polymers in the presence of hydrogen peroxide, whereas transglutaminase couples glutamine and lysine residues; both produce elastic and biodegradable networks whose modulus can be varied over an order of magnitude by adjusting the enzyme and substrate concentrations, although an exogenous protein is introduced that must subsequently be cleared [59]. The above gelation mechanisms are summarized in Table 2.

### 3.3. Network Parameters Governing Drug Release and Tumor Retention

The release behavior of an injectable depot is determined not only by the gelation chemistry but also by a group of measurable network parameters. Crosslinking density directly determines the average mesh size of the network. When the mesh size is much larger than the hydrodynamic radius of the loaded drug, as in the case of doxorubicin or paclitaxel entrapped in a loosely crosslinked polysaccharide network, the drug diffuses freely and the release follows Fickian behavior; in this situation, the extent of burst release mainly depends on the amount of drug distributed near the gel-tissue interface [80]. When the mesh size approaches the size of the cargo, which is the case for proteins, antibodies, and nanoparticles, diffusion is markedly hindered and release becomes degradation-controlled, so that the duration of drug exposure is determined by the erosion rate of the network rather than by the diffusivity of the drug [81]. This explains the two-stage release commonly observed in nanoparticle-loaded hydrogels, and also explains why encapsulating a hydrophobic drug into nanocrystals or nanoparticles before loading suppresses burst release more effectively than simply increasing the polymer concentration [82].

Swelling behavior further modulates these processes. A hydrogel that swells markedly after injection dilutes its own network, enlarges the mesh size, and accelerates drug release, whereas a hydrogel that deswells becomes denser and retards release. Porosity acts on a larger scale and is mainly relevant to cellular cargo. Pores of tens of micrometers are required for CAR-T or CAR-NK cells to proliferate within the depot and subsequently migrate into the tumor, so a network suitable for retaining small-molecule drugs is usually too dense for cells [83]. Mechanical strength should be matched to the implantation site rather than simply maximized. Breast tissue and the postoperative tumor bed are soft, and an excessively stiff depot causes mechanical mismatch, which promotes fibrous encapsulation and may itself hinder the transport of drugs and immune cells. Conversely, a hydrogel that is too weak is easily displaced by respiratory and postural movement and loses its retention advantage [84].

Rheological properties determine whether a formulation can be injected at all. A suitable depot should exhibit a storage modulus higher than its loss modulus over the physiological frequency range, pronounced shear thinning, a yield stress low enough to allow for extrusion through a 21–26 G needle under manual pressure, and rapid recovery of modulus after the shear is removed [85]. Shear thinning and yield-stress behavior also protect encapsulated cells, because a yield-stress fluid passes through the needle as a plug and the damaging shear and extensional stresses are confined to a thin layer near the needle wall. For this reason, supramolecular polymer-nanoparticle hydrogels preserve lymphocyte viability during injection much better than viscous polymer solutions of comparable modulus [86]. Degradation kinetics should likewise be matched to the therapeutic objective. A depot intended to cover the perioperative period of highest recurrence risk needs to remain intact for two to four weeks, whereas a depot intended to sustain an immune response requires an even longer retention time. However, most studies evaluate degradation only in PBS in vitro, and network parameters such as mesh size, yield stress, and swelling ratio are seldom reported, which makes direct comparison between different hydrogel systems difficult. Therefore, systematic characterization of rheological behavior, swelling ratio, degradation profile, and mesh size, together with the conventional cumulative release curve, provides a necessary basis for the rational design and comparison of injectable depots [87,88].

### 3.4. Drug Loading and Release Modes

Therapeutic agents are loaded through physical entrapment, electrostatic or affinity interactions, host–guest inclusion, or covalent conjugation. Physical entrapment is simple but prone to burst release when drug–network interactions are weak; electrostatic and affinity interactions improve retention of charged small molecules, peptides, proteins, and nucleic acid drugs (siRNA, mRNA, DNA); and covalent conjugation links the drug or prodrug to the backbone, enabling on-demand release through cleavage of acid-, enzyme-, redox-, or ROS-sensitive linkages [89]. The specific responsive and targeting designs built upon this material foundation are detailed in the following sections.

## 4. Tumor Microenvironment-Responsive Hydrogels

Among recent strategies for enhancing the precise and controlled release capability of injectable hydrogels, one approach exploits the sol–gel phase transition that hydrogels undergo in response to tumor microenvironmental stimuli (e.g., pH, enzymes, redox state, temperature) [90]. This transition gives rise to a stable three-dimensional network that serves as an in situ drug reservoir, thereby enabling sustained and slow drug release. The sections below follow the endogenous triggers in the order of the magnitude and reliability of the gradient they exploit—acidity, enzymatic activity, and reducing potential first, followed by the oxidative, hypoxic, and metabolic signals that are more variable—and close with exogenously triggered and multi-stimuli systems. Table 3 collects the comparison across all of these categories.

### 4.1. Hydrogels Responsive to the Acidic Tumor Microenvironment (pH)

Breast tumor tissue is generally characterized by aberrant glycolysis, local hypoxia, and the accumulation of metabolic products, which render the extracellular environment of tumor cells weakly acidic relative to normal tissue [91,92]. Consequently, acidic pH ranks among the most commonly utilized endogenous triggers when constructing locally responsive carriers aimed at breast cancer. In contrast to systemic delivery, pH-sensitive injectable hydrogels are able to establish a localized drug reservoir right at the tumor location or inside the post-surgical tumor cavity, where drug liberation is set off by the mildly acidic surroundings of the tumor. This enhances drug retention and utilization within the lesion while limiting exposure of off-target tissues [93,94]. Moreover, the extracellular pH (pH 6.5–6.9) of breast tumor tissue is considerably lower than the physiological pH 7.4 of normal tissue, providing the biological basis for constructing acid-sensitive hydrogels [95]. Capitalizing on this distinction from healthy tissue, researchers can engineer pH-sensitive hydrogels that, once within the acidic tumor region, experience charge inversion, protonation of pH-responsive moieties, or breakage of chemical linkages (for instance, boronate ester bonds, imine bonds), so as to govern drug release [96].

Among drugs used to treat breast cancer, raloxifene (RLF) can treat invasive breast cancer by selectively blocking the estrogen receptor (ER) [97]. However, oral RLF faces several challenges, including low solubility, limited targeting, and first-pass effects. To address these issues, Doaa S. Hamad et al. constructed an RLF-loaded injectable hydrogel based on chitosan and glyceryl monooleate (IPHRLI). IPHRLI exploits surface charge reversal and hydrogel swelling under acidic conditions to enhance the solubility, bioavailability, and local sustained-release performance of RLF [98]. After 8 h, IPHRLI still maintained a release of 75.41% compared with free RLF, and intratumoral bioavailability was significantly increased by 4.07-fold. This indicates that hydrogel delivery systems responsive to the acidic tumor microenvironment hold promise in sustained drug release.

A self-assembling bovine serum albumin (BSA) hydrogel served as the carrier for the prolonged delivery of doxorubicin (DOX), quercetin (QUE), and its dihydro counterpart taxifolin (TAX) [99]. Their findings revealed that the injectable pH-responsive BSA hydrogel, via regulated release, boosted the cytotoxic effect, antitumor potency, necrosis, and apoptosis induced by QUE, TAX, and DOX in TNBC cells, consequently curbing the growth and migration of breast cancer. Hence, developing pH-responsive BSA hydrogels constitutes a promising vehicle for controlled drug delivery, attaining gradual release together with extended therapeutic benefit.

Compared with the natural products described above used as hydrogel matrices, hybrid hydrogels have entered researchers’ field of view owing to their tunable mechanical and chemical properties and correspondingly higher drug-loading capacity [99]. Naved Malek et al. combined the CuBTC (HKUST-1) metal–organic framework with the synthetic polymer poly (vinyl alcohol) (PVA) to construct an injectable hybrid hydrogel loaded with 5-fluorouracil [100]. The results demonstrated that CuBTC, with its high surface area and porosity, significantly enhanced drug loading. A hydrogel composed entirely of PVA (12% w/v) without added CuBTC could encapsulate 5-fluorouracil at only 80 mM, whereas the hydrogel in which CuBTC (HKUST-1) and PVA acted synergistically reached an encapsulation capacity of 261.4 mM. Meanwhile, the previously added borax formed boronate ester bonds with the hydroxyl groups on the PVA chains; these bonds cleave under the acidic tumor microenvironment, enabling sustained and stable release of 5-fluorouracil. At pH 5.2, 5-fluorouracil release reached 15.4% in the first 6 h, 54.2% at 24 h, and 84.4% at 36 h. This study demonstrates that the acidic tumor microenvironment-responsive CuBTC–PVA hybrid hydrogel may serve as an ideal hydrogel for the long-term treatment of breast cancer.

**Figure 1:**
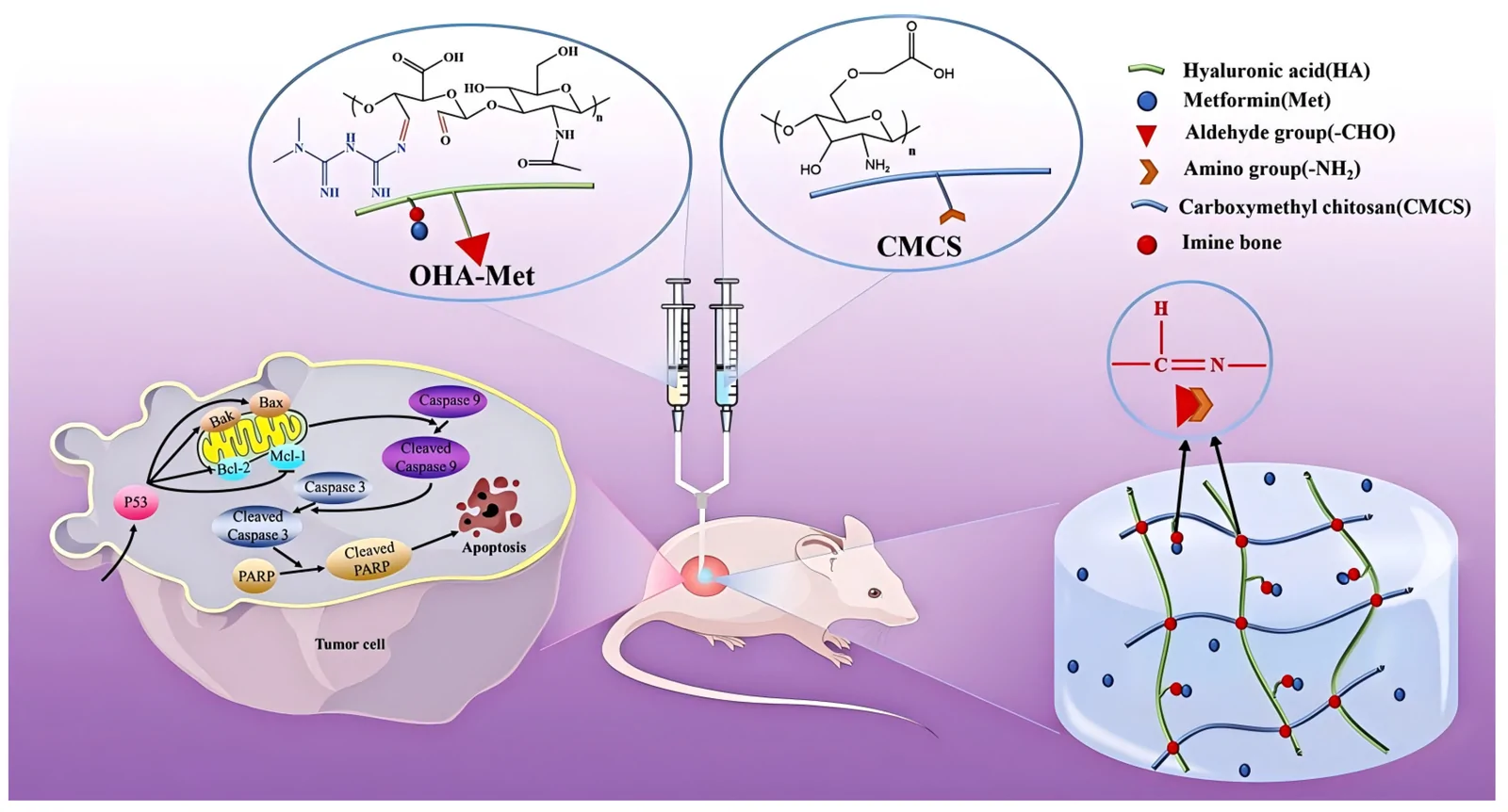
Schematic illustration of the injectable, acidic tumor microenvironment-responsive, metformin-loaded hyaluronic acid hydrogel (CMCS/OHA-Met) for inhibiting breast cancer recurrence [101].

In addition, dynamic covalent linkages offer another route toward pH-responsive platforms that combine improved degradability with tunable properties. Lihua Li and colleagues attached metformin (Met) onto oxidized hyaluronic acid (OHA) through imine linkages, after which the product was incorporated with carboxymethyl chitosan (CMCS) to generate a CMCS/OHA-Met hydrogel [101]. Their findings demonstrated that Met could be liberated from the CMCS/OHA-Met hydrogel under a mildly acidic condition of pH 5.5, thereby suppressing the recurrence of breast cancer. The detailed mechanism whereby the CMCS/OHA-Met hydrogel delivers metformin within the acidic tumor microenvironment to curb breast cancer recurrence is presented in Figure 1.

Hydrogels developed to respond to the acidic tumor microenvironment can exert sustained drug release and improve drug bioavailability by increasing drug encapsulation efficiency and permeability. However, the acidity of the tumor microenvironment is not uniform, and intratumoral heterogeneity exists; drug release rates differ under different pH values. Meanwhile, a single pH trigger is often insufficient to address the complex pathological features of breast cancer. Researchers have therefore continued to explore hydrogels with more specific responsiveness to the breast cancer microenvironment.

### 4.2. Hydrogels Responsive to the Enzymatic Tumor Microenvironment

The invasion and metastasis of breast cancer go hand in hand with remodeling of the extracellular matrix, which hinders the ability of drug carriers to penetrate the physical barrier and arrive at critical sites [102]. Yet this same process is accompanied by abnormally heightened activity of enzymes including hyaluronidase (HAase) and matrix metalloproteinases (MMPs). For this reason, engineering hydrogels that react to hyaluronidase and matrix metalloproteinases—enzymes distinguished by their capacity to break down the extracellular matrix—offers promise for building hydrogel systems geared toward accurate drug delivery.

HAase is a naturally occurring enzyme that catalyzes the hydrolysis of hyaluronic acid [103]. By degrading the physical barrier formed by the excessive accumulation of hyaluronic acid in tumor tissue, it further enhances drug penetration into the tumor [104]. In the work conducted by Lingfeng Xie and colleagues, an injectable hydrogel platform that integrated HAase with sodium alginate (ALG) was created to facilitate the diffusion of the naturally derived anticancer agent emodin (EMO), raising the regional level of EMO inside the tumor region and consequently enhancing therapeutic outcomes [105].

In addition, breast tumor tissue frequently overexpresses MMPs, which participate in degrading various protein components of the extracellular matrix (ECM) [106]. Hydrogels responsive to matrix metalloproteinases designed on the basis of this property can, with the aid of MMPs, enhance drug penetration into tumor tissue and thereby increase drug utilization [107]. Patients with TNBC need elevated doses of chemotherapeutic drugs and display resistance to chemotherapy; together with the heterogeneity of tumors and the absence of clearly defined molecular markers, their treatment becomes even more challenging, which results in pronounced systemic toxicity alongside restricted effectiveness [108]. Notably, the TNBC tumor region is distinguished by the upregulated expression of MMPs, especially MMP2 and MMP9 [109,110]. Such heightened levels of these enzymes create a chance to construct a responsive drug delivery platform capable of selectively liberating therapeutic agents once these enzymes are present, thereby optimizing treatment effectiveness while curbing systemic toxicity. Fu and co-workers devised a matrix metalloproteinase (MMP)-responsive hydrogel carrying sunitinib nanoparticles (NS-MRH) so as to improve radiosensitivity and consequently avert local recurrence of breast cancer [111]. In a separate investigation, Yang Lu and colleagues chemically linked a matrix metalloproteinase-2 (MMP-2) enzyme-sensitive peptide to a tissue-penetrating peptide, producing an MMP-2-responsive multifunctional peptide hydrogel system (aP/IR@FMKB) intended for photothermal-chemo-immunotherapy against breast cancer [112]. The aP/IR@FMKB assembly formed spontaneously from bufalin (BF), the photothermal compound IR820, and the immune checkpoint blocker aPD-L1, and was able to disassemble upon encountering MMP-2 enzyme to discharge its payload. Their findings additionally indicated that PD-L1 inhibition was reinforced via IR820 and ICD, markedly raising the abundance of tumor-infiltrating CD8^+^ T cells within both primary and distant tumors by factors of 3.5 and 5.2, respectively. This novel enzyme-responsive hydrogel strategy is capable of effectively reshaping the tumor immune microenvironment while showing responsiveness to PD-1/PD-L1 blockade. It further underscores the potential of this hydrogel within the realm of multimodal tumor treatment.

Hydrogels designed to respond to the enzymatic tumor microenvironment have greatly improved the precision of drug delivery to breast cancer tissue. Enzymatic triggers do, however, present a distinctive difficulty that pH triggers do not: MMP-2 and MMP-9 activity varies by more than an order of magnitude between breast cancer subtypes and between patients, it is regulated post-translationally by tissue inhibitors of metalloproteinases, and it is also elevated in the inflammatory tissue of a healing surgical wound—precisely the setting in which a postoperative depot is placed. Peptide-crosslinked gels are therefore susceptible both to premature erosion in an inflamed tumor bed and to under-release in a proteolytically quiescent lesion, and neither failure mode is detectable from the cumulative release curves usually reported [108]. However, owing to the rapid proliferation of tumor cells and the occurrence of metabolic abnormalities, not only are acidity and enzymatic activity elevated in the tumor microenvironment, but a vigorous oxidative stress response can also be triggered, manifested specifically as a marked increase in glutathione (GSH) levels. This feature offers an opportunity for the development of novel redox-responsive hydrogels.

### 4.3. Hydrogels Responsive to the Redox Tumor Microenvironment

Tumor cells generate large amounts of reactive oxygen species (ROS) owing to metabolic abnormalities and hypoxic stress [113]. To maintain redox homeostasis, intracellular GSH levels are markedly elevated (2–10 times higher than in normal tissue), forming a distinctive, highly reducing tumor microenvironment [114]. Within this redox environment created by high GSH, hydrogels covalently crosslinked by disulfide bonds are readily triggered, causing the disulfide bonds to cleave and thereby releasing drugs precisely into tumor tissue [115].

The abundant level of GSH within the tumor region serves as a key protective strategy through which malignant cells counteract oxidative stress, thereby furnishing a targeting foundation for engineering redox-responsive hydrogel delivery platforms. Rongzhan Fu and colleagues fabricated an injectable hyaluronic acid–tannic acid (HA-TA) hydrogel embedded with MXene@Cu nanosheets (HA-TA/MXene@Cu hydrogel) exhibiting anti-breast cancer activity [116]. Through the depletion of GSH, the HA-TA/MXene@Cu hydrogel converts the Cu^2+^ within the MXene@Cu nanosheets into Cu, which drives the Fenton reaction, resulting in the buildup of lipid peroxides (LPO) and triggering ferroptosis in cancer cells. At the same time, MXene exhibits outstanding photothermal conversion performance that enables photothermal therapy (PTT), allowing destruction of tumor tissue under near-infrared laser exposure. By this means, the ferroptosis induced by the redox-responsive hydrogel together with PTT operate cooperatively to produce a remarkably strong antitumor effect against breast cancer. This outcome consequently unlocks fresh possibilities for applying redox-responsive hydrogels in the treatment of breast cancer.

In addition, the supramolecular self-assembly strategy offers a new route for constructing redox-responsive hydrogels. Recently, the team of Debapratim Das developed an injectable hydrogel system based on a dipeptide gelator for the delivery of doxorubicin [117]. This hydrogel remains structurally stable in normal tissue; upon encountering the high-GSH environment of the tumor, the disulfide bonds within the hydrogel network are reductively cleaved by thiol groups, thereby releasing the drug. This dependence on a high GSH concentration achieves hydrogel stability in normal tissue and selective drug release at the tumor site. In vivo studies showed that a single injection shrank the tumor by approximately 75% within 18 days, with efficacy superior to the free drug and without systemic toxicity. The system achieves synergistic killing through locally high-concentration administration, induction of G2/M phase arrest, and activation of the mitochondrial apoptotic pathway. Furthermore, compared with metal-catalyzed Fenton systems, this purely GSH-responsive ultrashort peptide hydrogel avoids metal toxicity and exhibits superior biocompatibility and prospects for clinical translation.

The same disulfide chemistry can be moved from the network to the drug itself. Yuan and colleagues combined a prodrug strategy with bio-nanotechnology, conjugating epirubicin (EPI) to a C18 lipid chain through a disulfide bond and synthesizing three lipophilic prodrugs (α-ESC, β-ESC, γ-ESC) that differ only in the position of the disulfide bond, which were then formulated into carrier-free nanoassemblies (α-/β-/γ-ESC NAs) in order to overcome the short half-life and pronounced toxicity of EPI. In breast cancer models, drug release was triggered by the reductive, GSH-rich intracellular microenvironment of tumor cells, which cleaved the disulfide bond and thereby enhanced the tumor-directed delivery and controlled release of epirubicin [118]. Placing the redox-labile bond in a prodrug rather than in the crosslinker is attractive for hydrogel design because it decouples release chemistry from mechanical stability—the network can then be optimized for retention and injectability while the prodrug governs when the active species appears—and it offers a straightforward way to load a GSH-responsive payload into an otherwise inert thermogel. It also concentrates the response inside the cell, where the GSH gradient is genuinely steep, rather than in the extracellular space, where it is not; this distinction is frequently blurred in reports of “GSH-responsive” depots that are in fact bathed in an extracellular fluid of near-normal reducing potential [119].

### 4.4. Hydrogels Responsive to Reactive Oxygen Species

Whereas redox-responsive systems make use of the reducing capacity of the cytosol, ROS-responsive systems make use of the oxidative burden of the tumor interstitium, in which hydrogen peroxide accumulates to micromolar levels far above those of normal tissue. Several linker chemistries are commonly employed [120]. Thioketal linkages fragment upon oxidation; aryl boronic esters are oxidized to phenols and subsequently hydrolyze; thioethers undergo a hydrophobic-to-hydrophilic transition upon oxidation to sulfoxide without cleavage; and diselenide bonds are cleaved by both oxidants and reductants, so that they respond to either arm of the redox imbalance [121].

Yi and colleagues crosslinked aldehyde-functionalized hyaluronic acid with a thioketal-containing diamine, so that the resulting hydrogel degrades under both oxidative and acidic conditions, and used it to co-deliver 5-fluorouracil and the photosensitizer precursor 5-aminolevulinic acid [122]. In a breast tumor model, this dual-responsive hydrogel achieved sustained and stimulus-gated release, combined chemotherapy with photodynamic therapy, and simultaneously accelerated healing of the postoperative wound. In a related design, a ROS-cleavable thioketal is incorporated into a thermoresponsive PNIPAM network loaded with photosensitizer-conjugated gold nanorods. Singlet oxygen generated under light irradiation degrades the network that carries the photosensitizer, thereby forming a self-amplifying cycle in which therapy and drug release reinforce each other [123].

However, the concentration of endogenous hydrogen peroxide in breast tumors, although elevated, is usually insufficient to degrade a covalently crosslinked network within a therapeutically useful period. Most reported ROS-responsive hydrogels therefore rely on ROS generated exogenously by photodynamic or sonodynamic irradiation, or catalytically by an embedded Fenton agent [124]. Such systems provide reliable and operator-controlled release, but their selectivity derives mainly from the site of irradiation rather than from the biology of the lesion. Release under endogenous ROS levels alone therefore needs to be evaluated separately.

### 4.5. Hypoxia-Responsive and Hypoxia-Modulating Hydrogels

Severe hypoxia is a characteristic feature of breast tumors larger than a few millimeters and contributes to chemoresistance, radioresistance, and immune exclusion. Two opposite design strategies have been developed accordingly. In the first, hypoxia is used as the trigger: nitroimidazole and azobenzene groups incorporated into the network or into a prodrug linker are reduced by nitroreductases and azoreductases, whose activity increases markedly under low oxygen tension, so that the payload is released preferentially in the hypoxic core. In the second, hypoxia is regarded as an obstacle to be relieved, which is the more common approach in the hydrogel literature because many combination modalities depend on oxygen [125]. Hou and colleagues incorporated humic acid and MnO_2_ nanoparticles into an agarose hydrogel. The MnO_2_ component consumes intratumoral hydrogen peroxide to generate oxygen in situ, thereby relieving hypoxia and enhancing photodynamic killing, and the same reaction depletes GSH and releases Mn^2+^, which can serve as a magnetic resonance contrast agent [19].

Hypoxia-responsive injectable hydrogels designed specifically for breast cancer remain scarce, and most reduction-sensitive chemistry has so far been demonstrated at the nanoparticle rather than the network level. Azoreductase-mediated cleavage is slow relative to the period over which a depot must release its cargo, the enzymes involved are also expressed in gut microbiota and hepatic tissue, and oxygen tension varies steeply within a single lesion, so that a network designed to dissolve under hypoxia degrades unevenly [126]. At present, therefore, the hypoxia-relieving strategy, in which the depot normalizes the microenvironment for a partner modality, appears more practical than hypoxia-triggered release.

### 4.6. ATP- and Glucose-Responsive Hydrogels

Metabolic reprogramming provides two further triggers. Adenosine triphosphate is present in the tumor interstitium at concentrations far above those of normal tissue and reaches the low millimolar range within cells. It can be recognized in two ways: through an ATP-binding aptamer used as a crosslinker in a DNA hydrogel, in which target binding displaces the duplex and dissolves the network, or through the phenylboronic acid-ribose interaction, in which ATP competes with the diol crosslinks of a boronate ester network [127]. Glucose responsiveness is based on the same boronate chemistry or on glucose oxidase, which converts glucose into gluconic acid and hydrogen peroxide, thereby lowering the local pH and raising the local ROS level while consuming the fuel of the tumor [128].

A glucose oxidase-loaded depot therefore acts simultaneously as a therapeutic agent and as a generator of triggers. Lee immobilized glucose oxidase on a gelatin hydrogel by bioorthogonal click chemistry and implanted it at the surgical site in a breast cancer model. The immobilized enzyme retained its activity and continuously generated hydrogen peroxide, thereby suppressing tumor recurrence over time, whereas free glucose oxidase was rapidly cleared. Systemic absorption was minimal and the gelatin network was degraded within approximately two weeks [129]. In addition, the gluconic acid generated accelerates the hydrolysis of acid-labile linkages elsewhere in the network, and the hydrogen peroxide produced can feed a Fenton catalyst for chemodynamic therapy. However, neither ATP nor glucose is confined to tumor tissue, the extracellular gradients are modest, and the consumption of glucose and oxygen by an implanted enzyme also affects the surrounding normal tissue. These triggers are therefore more suitable for coupling drug release to metabolic activity within a lesion that has already been targeted physically by injection than for providing tumor selectivity by themselves [130,131].

### 4.7. Temperature-Responsive and Exogenous Hyperthermia-Triggered Hydrogels

Thermosensitive hydrogels are able to react to temperature cues that rise within the tumor because of metabolic or inflammatory activity. They additionally exhibit the feature of switching through a sol–gel phase change across a defined temperature window and are frequently applied to deliver a variety of anticancer drugs [132]. Such hydrogels remain liquid at lower temperatures and convert into a gel once the temperature climbs, notably when 37 °C is attained [133]. Owing to this sol–gel transition behavior, the hydrogel takes on a gel form within the warmer tumor tissue, which favors localized, prolonged, and gradual liberation of the therapeutic agent.

A composite platform pairing a thermosensitive hydrogel with a nanoparticle formulation represents an approach for boosting drug loading and preventing premature drug release. Manish Kumar and co-workers formulated an injectable hydrogel carrying paclitaxel nanocrystals (PTX-Lipid-NCs), which stays in a sol state at the reduced temperature of 2–8 °C and shifts into a gel state at body temperature, displaying good injectability together with a swift sol–gel transition [134]. Furthermore, in vivo experiments revealed that, relative to the hydrogel containing PTX alone, the PTX-Lipid-NCs hydrogel achieved superior efficacy in suppressing breast cancer.

In a separate investigation, an injectable thermosensitive hydrogel was employed to locally co-administer the ATR inhibitor BAY-1895344 (BAY) alongside doxorubicin (DOX), interfering with the DNA damage repair (DDR) pathway in malignant cells and improving chemosensitivity by a factor of eight relative to single-agent treatment. The injectable thermosensitive hydrogel DOX + BAY@Gel, created by Fengjiang Du and colleagues, underwent rapid gelation and established a stable gel network at 37 °C within a triple-negative breast cancer model, reaching a tumor suppression rate of 79.4%, which notably surpassed the 58% recorded for free DOX alone [135]. This twofold-action approach counteracts chemoresistance by shutting down the DDR compensatory pathway and extends the period of tumor suppression via regulated drug release. By merging stimulus-responsive drug delivery with disruption of the DDR pathway to achieve synergistic effects, this hydrogel system delivers a functional advance in localized combination therapy.

### 4.8. Multi-Stimuli-Responsive Hydrogels

The tumor microenvironment is highly complex and heterogeneous, and a hydrogel responsive to a single tumor microenvironmental feature is sometimes insufficient to achieve precise drug release. Therefore, multi-stimuli-responsive hydrogels—capable of responding simultaneously to two or more tumor microenvironmental features, or simultaneously to the tumor microenvironment and an exogenous stimulus—have become a research trend.

Mintao Liu’s group created an in situ injectable, temperature/pH dual-responsive hydrogel (FCPC) carrying a piperazine-functionalized celastrol derivative (PIPCel) [136]. This system is capable of swiftly establishing a stable hydrogel at 37 °C and discharging PIPCel when exposed to the acidic surroundings of the tumor (pH 6.5). In contrast to the 14% PIPCel release recorded under normal conditions, the release reached 76% within the acidic setting (pH 6.5). In addition, in vitro assays demonstrated that, absent acidic stimulation, the apoptosis proportion of 4T1 cells in the FCPC group stood at 19.88%, while the FCPC + Acid group reached 26.21%, markedly above that of the FCPC group, indicating that FCPC, by responding to the acidic tumor region, can substantially enhance breast cancer cell apoptosis and produce an anticancer effect. This dual-responsive hydrogel tackles the issues of poor aqueous solubility and limited bioavailability of PIPCel, accomplishing accurate and sustained drug delivery, exhibiting strong tumor-inhibitory performance in breast cancer treatment, and offering a fresh route for delivering poorly water-soluble natural anticancer agents. The applications of multi-stimuli-responsive hydrogels in breast cancer treatment are summarized below in Table 4.

**Table 3:** Comparison of endogenous and exogenous triggers used in injectable breast cancer hydrogels.

Trigger	Gradient in the Breast Tumor	Responsive Chemistry	Release Mechanism	Representative Therapeutic Performance	Principal Limitation
Acidic pH	Extracellular pH 6.5–6.9 versus 7.4; endolysosomal pH about 5.0–5.5	Imine, acylhydrazone, boronate ester, charge-reversal polymers	Protonation and hydrolysis of acid-labile bonds; swelling and charge reversal	Raloxifene-loaded invasome hydrogel retained 75.41% release at 8 h and raised intratumoral bioavailability 4.07-fold [98]; CuBTC-PVA hydrogel released 15.4%, 54.2% and 84.4% of 5-fluorouracil at 6, 24 and 36 h at pH 5.2 [100]	Contrast at extracellular pH is modest, and much reported selectivity is measured at lysosomal pH
Enzymes	MMP-2/MMP-9 and hyaluronidase overexpression, varying with subtype and patient	MMP-cleavable peptide crosslinkers (GPLGVRGK, PVGLIG); hyaluronic acid backbones	Localized proteolytic erosion at the gel-tissue interface; concurrent loosening of the ECM	aP/IR@FMKB peptide hydrogel increased tumor-infiltrating CD8^+^ T cells 3.5-fold in primary and 5.2-fold in distant tumors [112]; HAase-alginate hydrogel raised intratumoral emodin levels and deepened penetration [105]	Activity is also elevated in healing surgical wounds, and inter-patient variability is wide
Reducing potential (GSH)	Intracellular GSH 2–10 times that of normal tissue	Disulfide crosslinks; disulfide-linked prodrugs	Reductive cleavage of disulfide bonds after cellular uptake	Ultrashort peptide hydrogel shrank tumors by about 75% within 18 days after a single injection, without systemic toxicity [117]; HA-TA/MXene@Cu hydrogel depleted GSH and induced ferroptosis combined with PTT [116]	The steep gradient is intracellular, so extracellular depots respond weakly
ROS	Micromolar H_2_O_2_ in the interstitium, and far higher under PDT or SDT	Thioketal, aryl boronic ester, thioether, diselenide	Oxidative fragmentation of the linker; self-amplifying release when ROS are generated by irradiation	Thioketal-hyaluronic acid hydrogel gave stimulus-gated release of 5-fluorouracil and 5-aminolevulinic acid, combining chemo-photodynamic therapy with accelerated wound healing [122]	Endogenous levels are usually insufficient, and selectivity often derives from the irradiation field
Hypoxia	Low oxygen tension in the tumor core; elevated nitroreductase and azoreductase activity	Nitroimidazole and azobenzene linkers, or MnO_2_-mediated hypoxia relief	Reductive cleavage in the hypoxic core, or in situ oxygen generation for a partner modality	Humic acid/MnO_2_ agarose hydrogel generated oxygen in situ, depleted GSH and markedly enhanced photodynamic killing while providing MRI contrast [19]	Steep intralesional gradients cause uneven degradation, and the reductases are not tumor-exclusive
ATP and glucose	Interstitial ATP far above normal tissue; elevated glucose flux	ATP aptamer crosslinks; phenylboronate-diol exchange; glucose oxidase cascades	Competitive displacement of crosslinks; cascade generation of gluconic acid and H_2_O_2_	Glucose oxidase-immobilized gelatin hydrogel retained enzymatic activity at the surgical site and suppressed breast tumor recurrence with minimal systemic absorption [137]	The analytes are ubiquitous, and enzymatic consumption of glucose and oxygen also affects normal tissue
Temperature (endogenous)	About 1 °C above the surrounding tissue	Poloxamer, PNIPAM, PLGA-PEG-PLGA phase transition	Sol–gel transition at body temperature; subsequent diffusion- and erosion-controlled release	DOX + BAY@Gel achieved a tumor suppression rate of 79.4% in a TNBC model, compared with 58% for free DOX [135]; PTX-Lipid-NC hydrogel outperformed the PTX-only hydrogel [134]	Not tumor-selective, since the trigger is body temperature rather than the lesion
Exogenous stimuli (NIR light, alternating magnetic field, ultrasound)	Applied by the operator	Photothermal, magnetothermal or sonomechanical disruption of the network	On-demand, repeatable and quantifiable release	Magnetic supramolecular hydrogel underwent a magnetothermal gel-to-sol transition that filled irregular resection cavities and released PTX and DOX on demand, preventing recurrence [138]	Requires access to the lesion, and penetration depth limits light-based triggering
Multi-stimuli	Two or more of the above	Combined dynamic bonds, or endogenous plus exogenous gating	AND logic sharpens selectivity, whereas OR logic guarantees release	Temperature/pH dual-responsive FCPC hydrogel released 76% of PIPCel at pH 6.5 versus 14% under physiological conditions, and raised 4T1 apoptosis from 19.88% to 26.21% [136]	Rarely benchmarked against the corresponding single-responsive controls

**Table 4:** Applications of Multistimulus-Responsive Hydrogels in Breast Cancer.

Type of Stimuli	Hydrogel	Nanomaterial	Drug	Primary Function
PH+ Glutathione Responsive Hydrogel	IC1-R Peptide Hydrogel		Paclitaxel (PTX)	The pH/redox-responsive IC1-R-PTX hydrogel enables sustained PTX release and enhances breast cancer suppression [139].
PH+ Glutathione Responsive Hydrogel	DOX-DSNG	liposomes	DOX, dextran	Injecting composite hydrogels can continuously release anti-angiogenic and chemotherapy drugs, exerting anti-breast cancer tumor and anti-angiogenic effects [140].
Temperature- and Magnetically Responsive Hydrogel	Magnetic Supramolecular Hydrogels (MSH)	Polyethylene glycol-modified Fe_3_O_4_ nanoparticles	Paclitaxel and Doxorubicin	Injection of MSH triggers a magnetic-thermal gel-to-sol transition under an alternating magnetic field (AMF). The resulting sol conforms to irregular post-surgical cavities and enables heat-activated co-release of PTX and DOX, thereby preventing breast cancer recurrence [138].
Temperature- and Magnetically Responsive Hydrogel	Thermosensitive Magnetic Hydrogel	Chitosan, Fe^3+^ @DF-PEG-DFNPs	DOX + DTX	Alternating magnetic field (AMF) triggers a sol–gel transition via magneto-thermal effect. The dual-drug-loaded magnetic hydrogel (DDMH) shows enhanced drug release and superior therapeutic efficacy compared to single-drug PLGA-NP hydrogels [141].
Temperature- and Magnetically Responsive Hydrogel	DOX-GO/IONP/PEI-GEL	Chitosan	Doxorubicin (DOX)	The injected hydrogel responds to the tumor’s hyperthermic microenvironment, and its antitumor efficacy is significantly enhanced under an alternating magnetic field (AMF) [142].

Hydrogels engineered to react to the tumor region have substantially improved the accurate and prolonged liberation of drugs, contributing significantly to breast cancer therapy. Nevertheless, the actual TME constitutes an intricate, ever-changing, and non-uniform ecosystem. With the goal of more precisely targeting breast cancer cells and raising the efficiency and bioavailability of drug delivery, investigators have directed their focus toward particular molecules abundantly displayed on the membranes of tumor cells and, building upon this feature, have devised active ligand-functionalized hydrogels to refine hydrogel systems for the targeted, accurate delivery of drugs to breast cancer.

## 5. Active Ligand and Biofunctionalized Injectable Hydrogels

Although conventional systemic chemotherapy is the mainstay of breast cancer treatment, the off-target toxicity and dose-limiting toxicity caused by its nonspecific distribution severely constrain improvements in clinical efficacy [143]. To overcome this dilemma, active targeting strategies based on ligand–receptor recognition have emerged, in which specific ligands, peptides, or aptamers are used to modify the surface of drug carriers [144]. Through the selective interaction of ligands with receptors overexpressed on breast cancer cells (such as CD44 and HER2 receptors), specific recognition and enrichment in tumor cells are achieved [29,145]. To this end, advances in surface modification technology have enabled hydrogels to cross biological barriers and achieve precise delivery. In breast cancer, the aberrant expression of receptors such as CD44, the folate receptor, HER1, and CXCR4 provides diverse target options for ligand modification.

### 5.1. Small-Molecule- and Polysaccharide–Ligand-Functionalized Hydrogels

As active targeting moieties, small-molecule ligands offer significant advantages over macromolecular antibodies or peptides, including low molecular weight, simple conjugation chemistry, non-immunogenicity, low cost, and ease of modification [146]. Commonly used small-molecule ligands include vitamins (folic acid, FA) and polysaccharides (hyaluronic acid, HA) [147,148]. By specifically binding to receptors overexpressed on the surface of tumor cells—such as the folate receptor and CD44 receptor—these ligands initiate receptor-mediated endocytosis, promoting carrier internalization and delivering drugs precisely into the interior of tumor cells.

Currently, HA-based drug delivery formulations provide several benefits, including extended drug retention, an absence of immunogenicity, selective homing to tumors, lessened toxicity from chemotherapeutic agents, and gradual, prolonged drug liberation. Fu and colleagues fabricated a multifunctional nanocomposite hydrogel bearing HA on its surface; this work successfully produced a CS-EMO NPs/HAase@ALG hydrogel platform that merges the strengths of chitosan nanoparticle-based delivery, HAase-driven permeability enhancement, and the in situ injectability of the hydrogel. In a different report, an HA-modified hydrogel, through targeting the CD44 receptor that is abundantly present on cancer stem cells (CSCs), drove the conversion of CSCs into ordinary cancer cells so as to deplete CSCs and, when paired with photothermal therapy (PTT), curbed the recurrence and spread of breast cancer [149]. Xia Zhao and co-workers prepared an HA-modified composite hydrogel for local administration (HA-ATRA-AuNPs, HAAG) which, by specifically recognizing the CD44 receptor abundantly displayed on CSCs, gave a sustained release of all-trans retinoic acid (ATRA) and gold nanoparticles (AuNPs), inducing cell-cycle arrest and encouraging the additional differentiation of CSCs into normal cancer cells to clear CSCs; combined with the photothermal characteristics of the gold nanoparticles, it showed considerable promise for eradicating CSCs and suppressing tumor metastasis and recurrence [150]. Functionalized natural polysaccharides serving as hydrogel matrices enhanced the in vivo retention and stability of exosome-like nanoparticles. Fan Huaying and colleagues prepared a hydrogel composed of oxidized hyaluronic acid (OHA)–functionalized carboxymethyl chitosan (CMCS) (OHA-CMCS), which anchors exosomes isolated and purified from ginseng onto the hydrogel through noncovalent interactions (hydrogen bonds), preserving their architecture and biological function and thereby boosting exosome stability and enabling sustained drug delivery. Meanwhile, thanks to the dynamic Schiff base linkages present, the gel likewise exhibits pH responsiveness, allowing it to hold the exosomes at the tumor location and discharge them gradually. Additionally, with the aim of lowering regional relapse following breast cancer surgery, Huaying Fan and colleagues engineered a sunitinib nanoparticle–loaded hyaluronic acid–acrylate (HA-AC) hydrogel that reacts to matrix metalloproteinases to accomplish targeted therapy [151].

In addition to the polysaccharide hyaluronic acid used as a ligand for modification, small-molecule ligands such as folic acid enhance the penetration of drugs targeting tumor tissue by targeting the folate receptor overexpressed on breast cancer cells. Dae Hyeok Yang and colleagues formulated an injectable hydrogel constructed from folic acid/poly(ethylene glycol) (PEG) together with glycidyl methacrylate chitosan joined through β-cyclodextrin, which conveys paclitaxel (PTX) by way of folate receptor-mediated endocytosis at the membrane of breast cancer cells [152]. Beyond this, certain investigators developed an injectable, in situ gelling, thermosensitive hyaluronic acid-methane-g-poly(N-isopropylacrylamide) (HACPN) hydrogel for housing FA-conjugated graphene oxide (GO) (GOFA), aimed at the targeted transport of the chemotherapeutic compound doxorubicin (DOX) [153]. The work demonstrated that GOFA-DOX/HACPN reacts to pH signals inside the tumor, gradually liberating GOFA so as to home in on the folate receptor located on the tumor membrane and thereby accomplishing targeted DOX delivery. Relative to the free DOX group and the GOFA-DOX group, the GOFA-DOX/HACPN group markedly shrank tumor volume, indicating its promise for the targeted delivery of drugs to breast cancer tumors.

### 5.2. Protein-, Peptide-, and Antibody Fragment-Functionalized Injectable Hydrogels

Proteins and peptides, owing to their good biocompatibility, synthetic feasibility, low cost, and low immunogenicity, are frequently selected as ligands for modifying hydrogels [154]. Concurrently, peptide hydrogels show the capacity to convey low-dose drugs as well as protein therapeutics and to react readily to stimulus signals.

Chemokine delivery platforms built on hydrogels have emerged as a novel means of mitigating the side effects linked to the uncontrolled discharge of therapeutic agents within the TME. Chemokines, functioning as chemotactic cytokines, govern the migration and tissue infiltration of immune cells and are indispensable to immune system activity through the engagement of immune cells with particular receptors [155]. CCL25 represents a protein ligand able to bind the CCR9 chemokine receptor [156]. To date, tumor cells transduced with CCL25 have been demonstrated in TNBC to substantially boost the gathering of CD8^+^ T cells inside the tumor and to curb TNBC tumor expansion in vivo. Tianmeng Sun and colleagues developed a thermosensitive hydrogel that holds CCL25 [157]. Through attachment to the CCR9 receptor presented on the membrane of CD8^+^ cells, this hydrogel accurately steers CCR9-abundant CD8^+^ cells toward tumors. Such a process reshapes the TME, strengthening antitumor immunity and restraining the growth of triple-negative breast cancer tumors.

Moreover, a new lipopeptide, C16KTTβAH, has drawn notable interest as a self-assembling molecule that gives rise to a peptide-based hydrogel. Janne Ruokolainen and colleagues developed a C16KTTβAH peptide hydrogel [158]. Their findings indicated that C16KTTβAH selectively destroyed MCF-7 breast cancer cells and might function as a candidate agent for managing breast cancer. In a separate study, Yaping Li and colleagues devised an injectable supramolecular peptide hydrogel carrying abemaciclib and NLG919, assembled from a peptide–drug amphiphile, intended for the neoadjuvant immunotherapy of TNBC [159]. The hydrogel achieved a prolonged release of abemaciclib and NLG919. Abemaciclib triggered immunogenic cell death (ICD) in tumor cells, encouraged the maturation of dendritic cells (DCs), and steered monocytes toward differentiation into classically activated macrophages (M1). NLG919 was discharged from NLG-HKD upon cellular uptake and suppressed IDO-1-mediated Kyn production and Tregs, thereby easing the immunosuppressive microenvironment. Within the hydrogel, the two drugs operated cooperatively to strengthen the infiltration and functional activity of CD8^+^ T cells, thus offering an effective approach to treating TNBC.

In addition to chemokines and self-assembling lipopeptides, three receptor systems that are well established in breast cancer nanomedicine are gradually being introduced into injectable depots. Integrins of the α_v_β_3_ and α_v_β_5_ are upregulated on tumor neovasculature and on invasive breast cancer cells, and are recognized by the RGD motif and its cyclic and tumor-penetrating derivatives [160]. Because RGD is short, inexpensive, and readily conjugated to acrylate, maleimide, or aldehyde groups, it can be grafted onto essentially any of the networks listed in Table 1. Shan and colleagues conjugated cyclic RGD to solid lipid nanoparticles and reported reduced adhesion and invasion of αvβ3 overexpressing breast cancer cells; notably, although cyclic RGD prolonged retention of the nanoparticles at the tumor, it simultaneously increased uptake by the mononuclear phagocyte system and thereby reduced total tumor accumulation [57]. It should also be noted that RGD serves a second and quite different purpose in cell-laden gels, where it supplies the adhesion motifs that encapsulated T cells require in order to survive and proliferate within the matrix [161].

EGFR, also designated HER1, is the most attractive target in triple-negative disease, for which neither endocrine nor HER2-directed therapy is applicable. It can be addressed with cetuximab-derived fragments, affibodies, or the 12-mer peptide GE11 [162]. Li and colleagues assembled stearic acid-modified GE11 into a nanoplatform loaded with doxorubicin, which achieved high loading and sustained release and suppressed the proliferation of MDA-MB-231 cells through the EGFR-downstream PI3K/AKT pathway, so that the carrier itself contributed to the antitumor effect [58]. The transferrin receptor, which is upregulated to meet the iron demand of proliferating cells, provides a well-characterized endocytic route. Jadia and colleagues prepared transferrin-targeted polymeric nanoparticles for the phototriggered treatment of breast cancer, in which receptor binding promoted internalization before light-triggered release [163]. Antibodies and antibody fragments constitute a further category of ligand, and examples already exist within the systems discussed in this review: affibody-DNA hybrid strands have been used to direct gold nanoparticles to HER2-overexpressing breast cancer cells [28], and anti-CD276 single-chain variable fragments have been engineered onto biomimetic nanovesicles that were subsequently loaded into a thermosensitive hydrogel [164]. Combinations of two ligands have also been examined; anti-CD44 and EGFR dual-targeted solid lipid nanoparticles, for example, were developed to deliver doxorubicin to triple-negative breast cancer cells [165].

These ligand classes differ in their practical characteristics. Antibodies and antibody fragments provide the highest affinity but are expensive, carry a risk of immunogenicity, and are difficult to conjugate without partial loss of activity. Peptides are inexpensive, stable under the conditions of most gelation chemistries, and easy to control in terms of density, but they bind weakly and are susceptible to proteolysis in the MMP-rich environment for which these gels are designed. Small molecules such as folic acid are the least demanding, but the receptors they address are not strictly tumor-specific. Ligand density requires particular attention and is seldom optimized in hydrogel work: too low a density provides insufficient avidity, whereas too high a density causes the carrier to be captured by the first layer of receptor-positive cells it encounters, an effect known as the binding-site barrier, which reduces rather than improves penetration into the depth of the lesion. The main targets available for ligand modification in breast cancer are summarized in Table 5.

Protein/peptide-ligand-modified hydrogels hold promise for achieving ligand-directed tumor targeting, immunotherapy, and triggered release of therapeutic drugs.

### 5.3. Aptamer- and Nucleic Acid–Functionalized Hydrogels

Aptamers, single-stranded DNA or RNA oligonucleotides, are a new type of target molecules [176]. Compared to other tumor-specific ligands, their advantages include compact size, low synthesis cost, excellent chemical stability, low immunogenicity, and excellent tissue penetration, which allows for highly selective binding to target molecules.

The HER2 aptamer, serving as a ligand directed at overexpressed human epidermal growth factor receptor-2 (HER-2), holds the capacity to bind specifically to the membrane HER2 protein and may be applied to functionalize hydrogels so as to raise the effectiveness of targeted drug delivery [166]. Accordingly, for breast cancer subtypes that overexpress HER2, the HER2 aptamer accomplishes accurate targeting and improved treatment by identifying cell-surface receptors. Tingmei Chen and colleagues constructed a dual-targeting aptamer-decorated DNA hydrogel platform (DTA-H) to realize efficient, stable, and targeted drug delivery [167]. The HER2 aptamer together with the AS1411 aptamer were incorporated into DTA-H by means of DNA base complementary pairing, loaded with DOX, and conveyed in a targeted fashion to HER2-overexpressing breast cancer cells. The findings revealed that DTA-H was capable of transporting both the chemotherapeutic drug and the aptamer nucleic acid drug to target cells, prompting the breakdown of HER2 protein and, acting in concert with the chemotherapeutic drug DOX, restraining the growth of HER2-positive breast cancer.

Furthermore, the EpCAM aptamer is able to specifically identify and home in on tumor cells of epithelial lineage, such as breast cancer cells, fostering the buildup of nanodrugs at the tumor location and reinforcing immune cell infiltration, thereby playing a pivotal part in the targeted diagnosis of breast cancer [173]. Kaimin Wang and colleagues employed exosomes derived from breast cancer cells as a model target and devised a dual-aptamer (EpCAM-T2 and CD63-T1)-modified hydrogel for the effective separation and detection of breast cancer exosomes [174]. Notably, this work supplies an innovative and economical method for isolating and identifying tumor-derived exosomes. However, one potential limitation is that healthy epithelial cells expressing EpCAM could give rise to off-target effects, while the uneven expression of EpCAM across tumor cells may influence targeting accuracy.

Beyond the aforementioned aptamers used to functionalize hydrogels for the targeted treatment of breast cancers strongly expressing ER, PR, and HER2 receptors, for triple-negative breast cancer that lacks these receptors, alternative aptamer-modified hydrogels may likewise be applied to enhance the selectivity of the delivery platform. The LXL-DNA aptamer can effectively recognize triple-negative breast cancer MDA-MB-231 cells [177]. Its modified hydrogel is able to convey siRNA so as to contribute positively to cancer gene therapy, mitigating the instability and nonspecific biodistribution of siRNA delivery platforms in vivo together with their deficient targeting ability, and boosting gene-silencing efficiency [178]. In one investigation, Shusheng Zhang and colleagues created a novel RNA triple-helix hydrogel embedded with a CXCR4 siRNA duplex (suppressing a miRNA capable of activating the breast cancer tumor region) and functionalized with the LXL-DNA aptamer (apt-DNA-Chol) to effectively home in on breast cancer MDA-MB-231 cells [179]. The work demonstrated that, relative to free duplex siRNA, the LXL-DNA aptamer–modified RNA triple-helix hydrogel proved markedly more potent in lowering MDA-MB-231 cell viability and heightening cytotoxicity, as well as in effectively silencing miRNA gene expression. It was additionally shown that cells displayed a high uptake rate of the LXL-DNA aptamer–modified delivery platform, furnishing a highly specific and selective route for the gene therapy of TNBC.

### 5.4. Biomimetic Membrane-Coated Systems Within Hydrogel Depots

A different approach to molecular recognition dispenses with a synthetic ligand and coats the nanocarrier with a natural cell membrane, so that the entire surface proteome of the source cell is transferred to the particle. Erythrocyte membranes contribute CD47 and thereby reduce phagocytic clearance; platelet membranes confer affinity for damaged vasculature and for circulating tumor cells; leukocyte membranes carry adhesion molecules that favor transendothelial migration; and homotypic breast cancer cell membranes exploit the self-adhesive interactions that cause tumor cells to bind preferentially to their own kind, while at the same time supplying a full complement of tumor antigens for immune priming. Hybrid coatings extend this principle further. Wang and colleagues fused bacterial outer membrane vesicles with cancer cell membranes to coat sonosensitizer-loaded nanoparticles, thereby combining homotypic recognition with the innate immunostimulatory signals of the bacterial component, and reported improved control of breast cancer bone metastasis under ultrasound irradiation 32.

Embedding such biomimetic particles in an injectable hydrogel is advantageous for two reasons. The gel prevents the rapid washout that limits membrane-coated nanoparticles after intratumoral injection, and it protects the coating from the shear and dilution associated with systemic administration, which is where most membrane damage occurs. The anti-CD276 scFv-functionalized nanovesicles delivered from a thermosensitive hydrogel in Section 7.3 illustrate this combination [164]. The remaining difficulties are those of manufacture rather than of principle: membrane vesicles are difficult to characterize, their protein composition varies with the passage number and culture conditions of the source cells, the completeness of coating is hard to quantify, and a product derived from cancer cells raises regulatory questions concerning residual nucleic acid and oncogenic potential that a synthetic ligand does not.

### 5.5. Contribution and Limitations of Active Targeting in Locally Injected Depots

Active targeting was developed to solve a problem of biodistribution, since an intravenously administered carrier has to find the tumor among all other tissues. A hydrogel injected into or beside the tumor has already solved that problem geometrically. A ligand can therefore act only over distances of micrometers rather than centimeters, and three contributions remain open to it. It can promote cellular internalization of the released nanocarrier, retain that carrier within the lesion against interstitial convection, and direct the payload toward a particular subpopulation rather than the bulk tumor. The hyaluronic acid-modified depots that recognize CD44 on cancer stem cells are the clearest example of the third effect, and they show that ligand modification remains worthwhile even when the carrier is delivered locally [149,150].

Two consequences follow for experimental design. First, the appropriate control is the same depot carrying the same nanocarrier without the ligand. Comparison against free drug, or against an untargeted nanoparticle given intravenously, confounds the contribution of the ligand with the much larger contribution of local injection. Second, the receptors concerned are not tumor-exclusive. CD44 is expressed on normal epithelium and on hematopoietic cells, EpCAM on healthy epithelial tissue, and folate receptor-alpha in kidney and lung, where it is only weakly expressed in much of triple-negative disease. HER2 defines only a fifth to a quarter of breast cancers. Ligand selection should therefore be matched to molecular subtype rather than adopted by default, and receptor expression in the model used should be reported alongside a direct comparison between the targeted and untargeted depots.

Defining those subtypes is itself a computational problem. Li and colleagues clustered breast cancer samples by K-means on the principal components of gene expression, and weighted the genes within each cluster according to biological function, prognostic ability, and correlation with known disease genes. Integrating these weights into a fusion network yielded prognostic biomarkers specific to each cluster of patients [180]. Approaches of this kind provide a basis for matching a given ligand or trigger to a defined patient stratum.

Based on the above, in order to achieve sustained and stable drug release and improved drug utilization while precisely targeting tumor tissue for drug release and avoiding normal tissue damage or systemic toxicity, researchers have recently developed hydrogels that can both respond to the tumor microenvironment and actively target specific molecules on the surface of tumor cells. This multifunctional, synergistic drug delivery system, through dual precise control, demonstrates great potential in achieving efficient and low-toxicity cancer treatment.

## 6. Hydrogel-Mediated Delivery of Diverse Therapeutic Payloads

Injectable hydrogels have been used to deliver agents of very different kinds, from small-molecule cytotoxics to living cells. Each class imposes its own requirements on the network that carries it, and a chemistry that suits a small molecule may be unusable for a protein or a cell [181]. This section first summarizes the principal therapeutic agents used in breast cancer, the doses at which they are given, and the limitations of systemic administration that a local depot is intended to relieve. It then examines the constraints that each payload class places on gelation chemistry, drug loading, and retention, covering small-molecule chemotherapeutics, natural products, proteins and peptides, nucleic acids, antibodies, extracellular vesicles, gene-editing complexes, and living cells.

### 6.1. Therapeutic Agents Used in Breast Cancer and the Rationale for Local Delivery

Conventional regimens deliver large systemic doses on an intermittent schedule. Only a small fraction of each dose reaches the tumor, and the remainder is responsible for the toxicity that limits the dose. An injectable depot changes this balance. It places a much smaller absolute amount of drug at the lesion, maintains the local concentration over days to weeks, and lowers the plasma peak that drives systemic toxicity. Table 6 lists the principal agents discussed in this review, together with their usual clinical dose, the limitations of conventional administration, and the advantage that local delivery is expected to offer.

### 6.2. Constraints Imposed by Different Payload Classes

The agent determines what has to be delivered; the class to which it belongs determines what the network must provide. Table 7 summarizes the comparison.

Small-molecule chemotherapeutics are the least demanding chemically, since doxorubicin, paclitaxel, docetaxel, and 5-fluorouracil tolerate all of the mechanisms listed in Table 2. Their difficulties are physical. Hydrophilic agents diffuse out rapidly and produce a burst unless they are retained electrostatically, whereas hydrophobic agents are poorly soluble and load inefficiently. Both problems are addressed by converting the drug into a nanocrystal, micelle, liposome, or polymer conjugate before gel loading, which is why so many of the systems reviewed here are nanoparticle-in-gel rather than drug-in-gel formulations [185]. Natural products account for a large share of this literature, since poor solubility and rapid first-pass metabolism make them obvious candidates for local depots [186]. The pharmaceutical rationale is sound, but the pharmacological evidence is weaker. Potencies are usually micromolar, local concentration-time profiles are rarely measured, and comparison against a standard cytotoxic agent delivered from the same depot is seldom made, so the contribution of the compound cannot be separated from that of the delivery format.

Proteins and peptides impose a stability constraint that governs the gelation chemistry. Radical photopolymerization modifies methionine, cysteine, and tryptophan residues, Michael addition consumes free thiols, and Schiff base crosslinking consumes lysine amino groups [187]. Mild physical mechanisms are therefore preferred, and the chemokine, checkpoint antibody, and cytokine depots are almost all thermosensitive or supramolecular systems [188]. Retention is a second difficulty, since a globular protein diffuses out within hours unless it is anchored electrostatically or through affinity sequences. Nucleic acids are limited by degradation rather than by denaturation. Naked siRNA and mRNA are cleaved within minutes and cannot enter cells unassisted, so the network must hold a condensed complex rather than the free oligonucleotide. Structural DNA hydrogels are a special case in which the nucleic acid is both the network and the drug [189]. Antibodies lie between proteins and nanoparticles. Erosion-controlled release suits sustained checkpoint blockade, but they aggregate at interfaces and the local dose is limited by the volume that can be injected [190].

Extracellular vesicles are the most fragile payload. Their activity depends on an intact lipid bilayer and surface proteome, so only non-covalent networks are suitable, as in the ginseng-derived exosome depot and the exosome-capturing gel. Standardization remains the principal obstacle. Gene-editing complexes are the frontier and, at present, a gap in the breast cancer literature. Cas9 ribonucleoproteins are large, highly charged, and rapidly cleared, and require the condensation strategies used for nucleic acids [191]. Li and colleagues compared three injectable thermosensitive hydrogels differing only in surface charge. The positively charged gel gave higher cellular uptake, greater editing efficiency, longer tumor accumulation, and better antitumor efficacy, which indicates that network charge rather than network chemistry alone is decisive for this payload [172]. A local depot addresses two of the obstacles to editing solid tumors, namely short residence time and systemic exposure of a bacterial protein, provided that editing efficiency and off-target activity are measured in vivo. Living cells are the most demanding payload of all, and the requirements they impose on injectability, porosity, and network mechanics.

## 7. Injectable Hydrogel-Mediated Immunotherapy of Breast Cancer

Recent investigations have demonstrated that immunotherapy aimed at breast cancer is advancing swiftly, and injectable hydrogels have garnered widespread interest owing to their capacity to regulate local immunity for the purpose of strengthening systemic anticancer immune responses.

Directing drugs specifically toward the immune system represents a hopeful approach for the successful treatment of cancer. Nevertheless, present-day systemic immunomodulatory treatments are hampered by drawbacks including brief drug duration, weak targeting to the action site, and serious adverse effects [192]. Owing to their adjustable characteristics and varied biological functions, injectable hydrogels accordingly constitute a valuable platform for the localized delivery of immunomodulators and T cells, able to additionally reshape the immune microenvironment so as to recruit, activate, and expand the host’s immune cells, thereby reinforcing systemic immunity [193]. Such a pioneering intervention shows promise for tackling the issues of weak targeting and serious side effects inherent in drug delivery systems. Moreover, cancer vaccines, immune checkpoint inhibitors, and chimeric antigen receptor T-cell therapy stand as three principal strategies within cancer immunotherapy [194]. With this in mind, the present section goes on to examine approaches in which hydrogels function as delivery vehicles to transport cancer vaccines, immune checkpoint inhibitors, and CAR-T cells to the tumor location in order to produce novel immunotherapeutic effects.

### 7.1. Local Remodeling of the Immune Microenvironment

Before the individual immunotherapeutic strategies are considered, it is useful to set out the cellular events that a hydrogel depot can influence. Most of the systems described below act on more than one of these events.

Immunogenic cell death is usually the point of entry. Anthracyclines, certain CDK4/6 inhibitors, and photodynamic or photothermal injury cause dying tumor cells to expose calreticulin, release high-mobility group box 1, and secrete ATP. The tumor thereby becomes a source of both antigen and danger signal. A depot sustains this stimulus over several days rather than delivering it as a single pulse. In the MMP-2-responsive peptide hydrogel, immunogenic cell death induced by IR820 reinforced PD-L1 blockade and markedly raised CD8^+^ T-cell infiltration in both primary and distant tumors [112].

Dendritic cell activation follows. Local adjuvants such as TLR agonists, STING agonists, and mannosylated antigen constructs promote maturation and antigen presentation. These agents are responsible for much of the toxicity of systemic immunotherapy, so confining them to the lesion is particularly valuable. Zhang and colleagues delivered SN38 together with an OX40 agonist antibody from a syringeable depot after breast tumor resection. The hydrogel activated the STING pathway in situ and increased the proportion of mature dendritic cells [195].

Macrophage repolarization is a third axis. Tumor-associated macrophages of the M2 phenotype suppress T-cell function and promote angiogenesis, and their abundance correlates with poor prognosis in breast cancer. Conversion toward the M1 phenotype is a recurrent readout in hydrogel studies. It has been achieved with TLR agonists, with metal ions such as Mn^2+^, and by depleting the M2 population outright, as in the mannosylated vaccine hydrogel [196]. In the depot mentioned above, the proportion of M1 macrophages rose while that of M2 macrophages fell [195]. The supramolecular peptide hydrogel likewise steered monocytes toward M1 differentiation.

T-cell recruitment is the fourth axis. Chemokine gradients established by a depot draw effector cells into a tumor that would otherwise exclude them. The CCL25-loaded thermosensitive hydrogel is the clearest example. By binding the CCR9 receptor, it directs CCR9-positive CD8^+^ cells toward the tumor and restrains the growth of triple-negative breast cancer [157].

Cytokine delivery supports these cells once they arrive. Systemic administration of interleukin-2, interleukin-12, or interferon-alpha is limited by severe toxicity, and the tolerated doses rarely achieve useful intratumoral concentrations. A local depot reverses this relationship by confining the cytokine to the lesion and prolonging its exposure. Interleukin-15 released from a polymer-nanoparticle hydrogel is the best-characterized example. It is discussed together with CAR-T delivery in Section 7.4, since the same principle applies whether the responding lymphocytes are endogenous or adoptively transferred [168].

What distinguishes the hydrogel approach is therefore not a new immunological mechanism. It is the pharmacokinetics of exposure, namely a sustained and high local concentration of agents whose systemic use is limited by autoimmune and inflammatory toxicity. The corresponding limitation is equally structural. A depot can remodel only the lesion that it occupies. Activity against distant and micrometastatic disease depends on whether local priming generates a systemic response, and the effects observed in distant tumors in bilateral models provide the main evidence that it does.

### 7.2. Hydrogel Cancer Vaccines

**Figure 2:**
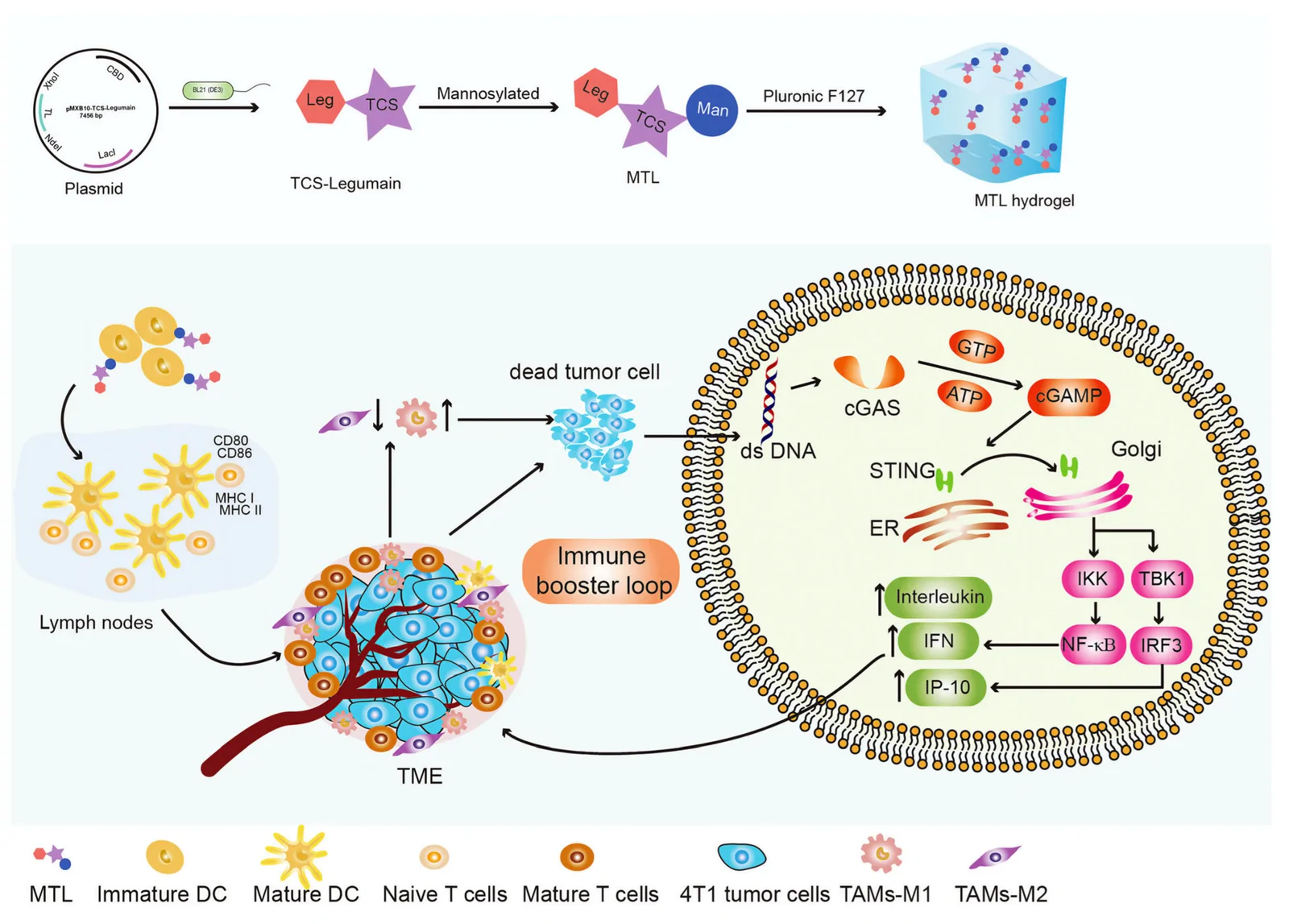
Mannose-functionalized hydrogel-based cancer vaccine promotes DC maturation and antigen presentation and induces CTL migration into the tumor, thereby depleting M2-type TAMs and remodeling the immune microenvironment to inhibit breast cancer growth [196].

Cancer vaccines have attracted broad attention in the early stage of the cancer-immunity cycle by eliciting sustained antitumor immune responses and durable tumor regression [197,198]. Yongzhuo Huang et al. utilized mannose to functionalize a genetically engineered protein vaccine made up of riboflavin (serving as adjuvant) and a legumain domain (acting as peptide antigen), then incorporated this vaccine into a hydrogel so as to home in on dendritic cells (DCs) [196]. The study revealed that the mannose-functionalized hydrogel cancer vaccine fostered DC maturation and antigen presentation and prompted the trafficking of CTLs into the tumor, thus restraining the growth, relapse, and spread of 4T1 breast tumors by eliminating M2-type TAMs and reshaping the immune microenvironment (Figure 2). Building on this, bioengineered hydrogel scaffolds incorporating cancer vaccines display strong potential as candidate tools for precision immuno-oncology.

### 7.3. Immune Checkpoint Blockade Therapy

Immune checkpoint inhibitors (ICIs) constitute a hopeful therapeutic route for treating breast cancer [199]. Available antibodies directed against PD-1, PD-L1, and CTLA4, acting as ICIs that block the corresponding receptors on the membrane of breast cancer cells, elicit antitumor immunity. Of these, programmed death-ligand 1 (PD-L1) is displayed at heightened levels on the membrane of TNBC cancer cells [200,201,202]. For this reason, the monoclonal antibody anti-PD-L1 (aPD-L1) is broadly employed to inhibit the overexpressed PD-L1 present on TNBC cells. Wenwen Huang and colleagues fabricated an injectable hydrogel composed of cysteine-tagged silk-elastin-like proteins (cSELPs) crosslinked with gold nanoparticles (AuNPs) for the in situ delivery of doxorubicin (DOX) together with the immune checkpoint inhibitor aPD-L1, producing combined photothermal and chemo-immunotherapy, interrupting the PD-1/PD-L1 interaction, effectively switching on antitumor immunity, and generating an anti-breast cancer effect [182]. This likewise unlocks a fresh pathway for hydrogel delivery platforms designed from modified proteins.

However, the clinical response to these inhibitors remains unsatisfactory. Therefore, Jin Sun et al. developed an injectable hydrogel scaffold that, through the local delivery of the small-molecule inhibitor PQ912 [203]. This suppresses the expression of CD47 on the surface of tumor cells, thereby blocking the CD47–SIRPα axis, enhancing the recognition and clearance of cancer cells by macrophages and antigen-presenting cells, activating the immune system, and exerting an anti-breast cancer effect.

**Figure 3:**
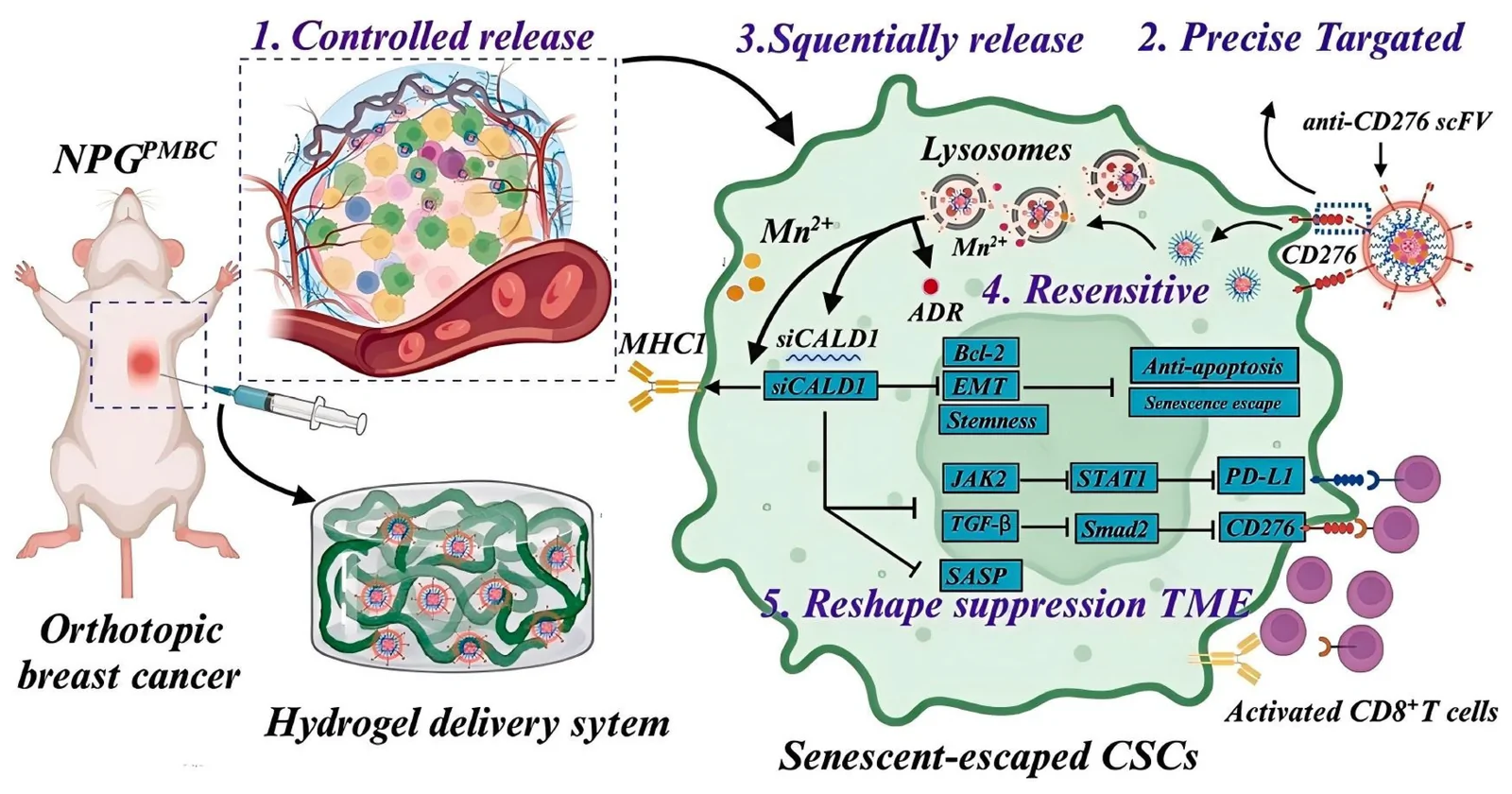
Controlled-release thermosensitive hydrogel housing anti-CD276 scFv–engineered biomimetic nanovesicles, which selectively merge with the cell membrane of senescence-escaping CSCs and sequentially discharge siCALD1 and ADR so as to produce immunotherapeutic effects [164].

Furthermore, in anthracycline (ADR)-resistant BC, the elevated expression of CD276 and PD-L1 alongside diminished MHC I permits senescent cancer stem cells (CSCs) to escape the immune surveillance implicated in BC onset, relapse, and spread. For this reason, Yourong Duan et al. devised a controlled-release thermosensitive hydrogel carrying pH-responsive anti-CD276 scFv–engineered biomimetic nanovesicles to overcome primary, recurrent, metastatic, and distant BC in humanized mouse models [164]. The anti-CD276 scFv–coated nanovesicles selectively merged with the cell membrane of senescence-escaping CSCs and then, owing to their pH responsiveness, sequentially released siCALD1 and ADR. siCALD1 in combination with ADR effectively triggered CSC apoptosis, lowered CD276 and PD-L1 expression, and boosted MHC I; together with Mn^2+^, it surmounted chemoresistance and enhanced CD8^+^ T-cell infiltration (Figure 3). This combination treatment strategy sheds light on how senescence-escaping CSCs dodge immune surveillance and furnishes a promising means of improving the effectiveness of immunotherapy in cancer treatment.

Based on the above, injectable hydrogels delivering immune agents play an important role in activating the immune system and reducing immune escape. The strength of the local approach is most evident for checkpoint antibodies, whose systemic administration causes immune-related adverse events in a substantial minority of patients while achieving only limited intratumoral concentrations. A depot placed in the tumor bed reverses that ratio. Two questions nevertheless remain open. The first is dose: the amounts of antibody that can be loaded into an injectable volume are small compared with a systemic course, and whether sustained local exposure at these levels is pharmacologically equivalent has not been established in any comparative study. The second is the site of action—PD-1/PD-L1 blockade acts substantially in the draining lymph node as well as in the tumor, so a depot that confines the antibody to the lesion may forfeit part of the mechanism it is intended to exploit. Studies that measure antibody concentration in the draining node, and that compare local depot against systemic dosing at matched total dose, would settle both points.

### 7.4. Chimeric Antigen Receptor T-Cell Therapy

Within breast cancer immunotherapy, integrating chimeric antigen receptor (CAR) technology demonstrates striking potential, notably CAR-modified T cells for the treatment of breast cancer [204]. T cells represent a key element of the immune system and hold the capacity to nonspecifically recognize and destroy tumor cells [205]. By means of genetic engineering, CAR constructs aimed at particular breast cancer antigens (such as Trop2, HER2, and EGFR) are incorporated into T cells, thereby strengthening their tumor-recognition ability and lessening cytotoxicity [206]. Functioning as carriers, hydrogels are able to prolong the survival of CAR-T cells within the tumor region so as to boost therapeutic outcomes.

Within the breast cancer tumor microenvironment, leakage of blood vessels and structural irregularities, coupled with raised tumor interstitial pressure, diminish the infiltration of CAR-T cells into solid tumors, signifying that the entry and proliferation of CAR-T cells within solid tumors encounter obstacles [207]. Administering CAR-T cells through a hydrogel carrier can substantially heighten tumor penetration and antitumor potency. Recent work has revealed that trophoblast cell-surface antigen 2 (Trop2), as a newly recognized tumor marker, is abundantly displayed on the membrane of TNBC [208]. Consequently, T-cell therapy directed at the Trop2 chimeric antigen receptor may act as a candidate for managing TNBC. Huadong Zhao and colleagues devised an injectable hydrogel to convey CAR-T cells along with the mitophagy agonist BC1618, which markedly bolstered the antitumor response of CAR-T cells by stimulating mitophagy, thereby alleviating TNBC [209]. The localized delivery via the injectable hydrogel produced a setting of intensified inflammation and mitophagy at the tumor location, thus driving CAR-T cell proliferation and maintaining antitumor activity to enhance therapeutic efficacy. The combination of BC1618 and Trop2-targeting CAR-T cells delivered via the hydrogel provides a promising strategy for the immunotherapy of TNBC. Additionally, drawing on the overexpression of epidermal growth factor receptor (EGFR, also termed HER1), this receptor has served as a target for designing novel targeted therapies against triple-negative breast cancer [170]. Hence, engineered HER1-CAR-T cells offer a hopeful strategy for treating HER1-positive TNBC tumors. However, tumor hypoxia and abnormal tumor vasculature dampen the viability and function of CAR-T cells, hindering HER1-CAR-T cells from reaching the anticipated efficacy in TNBC treatment [210]. To address this, Liangzhu Feng and colleagues created an injectable hydrogel (TMP@PEGgels) that reacts to endogenous H_2_O_2_ and is loaded with tetramethylpyrazine (TMP); through relieving tumor tissue hypoxia and remodeling the vasculature, it fosters the survival and tumor infiltration of HER1-CAR-T cells, thereby suppressing HER1 receptor–overexpressing MDA-MB-468 and MDA-MB-231 cells [171]. Relative to conventional intravenous administration of HER1-CAR-T cells and of free TMP plus HER1-CAR-T cells, the concurrent injectable administration of TMP@PEGgels and HER1-CAR-T cells brought about localized tumor infiltration of HER1-CAR-T cells. This work illustrates the capacity of the TMP-loaded hydrogel to improve the targeted therapeutic effectiveness of HER1-modified CAR-T cells against breast cancer tissue by easing tumor tissue hypoxia. In a comparable manner, natural killer (NK) cells engineered via CAR technology to recognize TNBC surface antigens (Trop2, HER2, and EGFR) likewise function to strengthen CAR-NK infiltration into tumor tissue and targeted therapeutic efficacy [211,212].

Cells are the most demanding cargo that a hydrogel can carry, and several technical obstacles remain. The first is viability during injection. Lymphocytes suspended in a viscous polymer solution are exposed to shear and extensional stresses within the needle that damage the membrane. A yield-stress, shear-thinning network avoids this problem, because the cell-laden material travels through the needle as a plug and the damaging stresses are confined to a thin layer at the wall. Grosskopf and colleagues exploited this behavior in a supramolecular polymer-nanoparticle hydrogel prepared by simple two-syringe mixing. The material was extruded through a 26 G needle with the cells intact and self-healed immediately afterwards [145]. The second obstacle concerns hydrogel-cell interactions. Encapsulated T cells require adhesion motifs such as RGD in order to survive and proliferate, and matrix stiffness and stress relaxation influence their activation and differentiation. Porosity must also permit proliferation within the depot and subsequent egress into the tumor. These requirements conflict, since a network open enough for cells to leave also loses its cytokine cargo rapidly, and the balance is at present established empirically. The third obstacle is the control of cell release. A cell must leave the depot in a viable and activated state, and at a rate matched to the persistence of the antigen. Release that is too rapid reproduces the outcome of a bolus injection, whereas release that is too slow strands the cells in a depot that is itself becoming hypoxic and acidic.

Immune persistence is a fourth consideration. The rationale for the hydrogel niche is that sustained local exposure to stimulatory cytokines expands the transferred cells in situ and preserves a less differentiated memory phenotype. Co-delivery of interleukin-15 from the polymer-nanoparticle hydrogel produced exactly this effect, while confining a cytokine that is poorly tolerated when given systemically. Whether the same construct can prevent the exhaustion caused by chronic antigen exposure in an immunosuppressive breast tumor has not yet been tested. Clinical translation is the final and least discussed obstacle. A cell-laden depot must be formulated at the bedside, within the viability window of the cell product and under sterile conditions. It also introduces a combination-product regulatory pathway in which the biomaterial and the cell therapy are assessed together, and it requires image guidance for accurate placement. Batch-to-batch variability and the cost of scale-up remain unresolved, and no hydrogel-delivered CAR-T or CAR-NK product has so far entered clinical evaluation [170,171]. The gap between the elegance of the preclinical constructs and the practicalities of manufacture appears to be the principal reason.

Based on the above, injectable hydrogels play an important role in delivering CAR-T cells bearing surface-modified antibodies and in enhancing their infiltration into breast cancer and targeted therapy.

## 8. Injectable Hydrogels for the Diagnosis and Multimodal Combination Therapy of Breast Cancer

Injectable hydrogels are of considerable value in both the early detection and the management of breast cancer. Since their hydrophilic, three-dimensional crosslinked polymer framework closely mimics biological tissue, they can be paired with biosensors to attain swift, efficient, specific, and exceptionally sensitive identification of breast cancer by sensing early breast cancer biomarkers. This carries favorable implications for the early detection and treatment of breast cancer. In particular, within the developing field of immunotherapy, by means of functionalized delivery vehicles, they accomplish the targeted transport of immunosuppressants or T cells to tumor tissue, reinforce immune cell infiltration, and thereby deliver effective immunotherapy.

### 8.1. Injectable Hydrogels for the Diagnosis of Breast Cancer

Early breast cancer detection is essential for effective treatment, but current methods have limitations. As a novel biomaterial, hydrogels offer a promising solution for developing highly sensitive biosensors for breast cancer detection.

At present, by combining a peptide hydrogel with the stable conductive polymer PEDOT, a new, ultrasensitive, antifouling electrochemical biosensor has been built for sensing the breast cancer biomarker HER2 [213]. In addition, in the course of tumor progression, reactive oxygen species (ROS)/reactive nitrogen species (RNS) are capable of upsetting the redox balance, modifying the intracellular and extracellular microenvironment, and pushing cells toward malignant transformation. For this reason, in order to track in real time the tight link between the ROS and RNS generated by 3D-cultured breast cancer cells subjected to drug stimulation and the malignant transformation of cells, Wensheng Yang and colleagues devised an antifouling peptide hydrogel electrochemical biosensor [214]. By engineering and encoding the amino acid sequence of synthetic Fmoc short peptides, they produced an Fmoc-FF:Fmoc-K(Fmoc)-D composite hydrogel, which effectively reproduced the extracellular matrix of three-dimensional cell culture and established a hydration barrier within the intricate biological setting to withstand biofouling of the sensing interface. The findings indicated that the rise in ROS/RNS was directly tied to the metastatic capacity of tumor cells, and that RNS might function as a pivotal contributor to cancer metastasis [215]. In a separate study, Abdollah Salimi and team members fabricated a fluorescent hydrogel by way of a Schiff base reaction occurring between the amino groups of chitosan and the aldehyde groups of carbon dots (CDs) for the sensitive measurement of the breast cancer biomarker miRNA-21 in MCF-7 cancer cells, thereby enabling early breast cancer detection [216]. This study revealed that the fluorescent hydrogel exhibits low cytotoxicity and may act as a biosensor for sensing early breast cancer biomarkers, which carries favorable value for the early detection of breast cancer.

However, hydrogel systems combined with biosensors for breast cancer diagnosis are still being refined, with the aim of maximizing the targeting rate.

### 8.2. Imaging-Integrated Theranostic Hydrogels

Biosensing addresses detection outside the body. A separate line of work embeds contrast agents in the depot itself, so that the construct that treats the lesion can also be visualized within it. Four imaging modalities are used, and they differ in the information they provide. It is worth noting that many of the components already described in this review serve this purpose without modification, since photothermal agents are also optical absorbers and magnetic nanoparticles are also contrast agents.

Fluorescence imaging is the most accessible modality. Near-infrared dyes such as IR780, IR820, and indocyanine green are already present in many photothermal depots as the photosensitizer, so imaging capability is obtained without adding a further component. The chemo-photothermal system described in Section 8.3 is an example, since IR780 is both the heat source and the fluorophore [128]. The pH-responsive Pt-TiO_2_ depot of Section 8.3 likewise carries a fluorescent probe and thereby reports its own distribution [148]. Penetration is limited to a few millimeters. Fluorescence is therefore restricted to superficial lesions, to the resection bed, and to intraoperative guidance, where it is genuinely useful for delineating margins.

Photoacoustic imaging converts the same optical absorption into an acoustic signal. It reaches centimeter depths with high spatial resolution. The gold, bismuth, polydopamine, and MXene components used for photothermal conversion are all effective photoacoustic contrast agents, so this capability is also obtained without redesigning the depot [174]. Wu and colleagues embedded gold nanorods labeled with iodine-125 and modified with an RGDY peptide in an injectable double-network hydrogel. The construct was injected into the resection cavity, where it combined photothermal therapy with brachytherapy and prevented local recurrence 32. Gold nanorods of this kind provide both photoacoustic and computed tomography contrast.

Magnetic resonance imaging offers unrestricted depth and good soft-tissue contrast, which matters for the breast. It is obtained by incorporating iron oxide nanoparticles for T2 contrast or manganese species for T1 contrast. Manganese-containing systems are attractive because the same ion serves several purposes. MnO_2_ consumes peroxide to relieve hypoxia and depletes GSH, and the Mn^2+^ released provides contrast and activates the cGAS-STING pathway, as described in Section 4.5 [143]. Shu and colleagues combined Prussian blue with manganese in a plug-and-play injectable hydrogel that supported photothermal treatment and immune activation while remaining visible on magnetic resonance imaging [149]. Computed tomography relies on elements of high atomic number, such as gold, bismuth, or tantalum, and is the natural choice when the depot must be localized relative to bone or when treatment planning for radiotherapy is required. Multimodal constructs combine two or three of these modalities, most often photoacoustic imaging with magnetic resonance imaging, in order to exploit their complementary resolution and depth.

An MRI-visible hydrogel requires a label, since the high water content of the network gives it relaxation properties close to those of the surrounding fluid, and the label is introduced either by attaching a paramagnetic chelate to the polymer backbone or by replacing gadolinium with a metal that the body already handles. Said and colleagues coupled a gadolinium chelate to hyaluronic acid and obtained an injectable, self-healing hydrogel that was detectable by T1 measurement in vivo, which allowed both the distribution and the degradation of the depot to be followed without repeated tissue sampling [217]. Rizzuti and colleagues assembled multicomponent peptide scaffolds with iron(III) and reported enhanced MRI detection, thereby avoiding the retention concerns associated with gadolinium chelates [218]. Both approaches are transferable to a breast cancer depot, where an MRI-visible gel would confirm that the resection cavity has been filled, allow erosion to be monitored during adjuvant radiotherapy, and help to distinguish residual gel from seroma or recurrent tumor at follow-up. Two reservations should be recorded. The first concerns purpose. In almost all published work the imaging component acts as a distribution tracer. It shows where the gel is and how it degrades, but it is not a measurement on which a clinical decision would be based. Genuine theranostic operation would require the signal to report drug release or therapeutic response quantitatively, and to do so with a calibration that holds in vivo. Very few systems have attempted this. The second reservation concerns safety. Gadolinium chelates carry a recognized risk of nephrogenic systemic fibrosis and are retained in tissue, manganese is neurotoxic on chronic exposure, and high-atomic-number nanoparticles are cleared slowly if at all. These considerations weigh more heavily in a depot that persists for weeks than in an intravenous contrast agent that is excreted within hours, and long-term clearance data are largely absent from this literature.

### 8.3. Multimodal Combination Therapy Hydrogels

Although immunotherapy is attracting increasing attention, chemotherapy remains one of the most effective means of treating cancer at present; however, chemotherapeutic drugs have shortcomings such as substantial toxic side effects and a tendency to develop drug resistance [219]. Compared with conventional single-treatment approaches, multimodal combination therapy demonstrates favorable therapeutic efficacy by integrating multiple single therapies. At the same time, if a nanomaterial-based hydrogel delivery system is used in treatment and further functionalized to confer characteristics such as targeting and controlled release, it can improve drug stability and bioavailability, holding great potential for enhancing cancer treatment efficacy.

The multimodal treatment modality combining chemotherapy and photothermal therapy can achieve maximal synergy in tumor treatment. For example, photothermal therapy (PTT) can accelerate the penetration of chemotherapeutic drugs at the tumor site and their intracellular delivery into cancer cells [220]. In recent years, nanocarrier-driven chemo-photothermal therapy (chemo-PTT) has proven to exhibit considerable application potential in oncological treatment. Nonetheless, the uptake of systemically delivered nanocarriers by tumor tissue falls below 1% [221]. To this end, the development of injectable hydrogels to deliver nanocarriers loaded with near-infrared fluorescent dyes and chemotherapeutic drugs directly to the tumor site has become a chemo-photothermal therapy with synergistically enhanced cancer treatment efficacy. Duarte de Melo-Diogo and colleagues created an in situ gelling, ionically crosslinked chitosan-based injectable hydrogel embedding nanoparticles loaded with the near-infrared fluorescent dye IR780 (IR/BPN) along with nanoparticles loaded with doxorubicin (DOX) (DOX/TPN) for synergistic chemo-photothermal treatment of breast cancer [183]. In vivo experiments demonstrated that, under near-infrared light exposure, the developed IR/BPN + DOX/TPN@Gel discharged 1.7 times more DOX than IR/BPN@Gel by itself. Meanwhile, in vitro experiments established that near-infrared light exposure of this hydrogel amplified its performance in the combined chemo-photothermal therapy of breast cancer. When IR/BPN + DOX/TPN@Gel was employed solely to convey the chemotherapeutic drug (DOX) without near-infrared light exposure, breast cancer cell viability was observed to drop to 85%. When only near-infrared light irradiation (photothermal therapy) was applied to IR/BPN@Gel, breast cancer cell viability decreased to 35%. Of interest, when IR/BPN + DOX/TPN@Gel delivered the chemotherapeutic drug DOX while near-infrared light irradiation was simultaneously applied, cancer cell viability decreased to approximately 9%, demonstrating the potential of the synergistic chemo-photothermal therapy of this hydrogel against breast cancer. From the above data, the synergistic therapy not only enhanced the drug-delivery capability of the hydrogel but also strengthened its inhibitory effect on cancer cells. A further study indicated that hydrogels designed for synergistic chemo-photothermal therapy are able to react to the acidic tumor region and serve a highly meaningful function in breast cancer relapse and dissemination. Yongli Shi et al. developed an injectable hydrogel (DCGD) made up of chitosan (CS), β-glycerophosphate (β-GP), and dopamine (DA) and incorporating DOX, which possesses near-infrared (NIR) photothermal characteristics together with a chemotherapeutic drug, so as to deliver synergistic chemo-photothermal therapy for averting breast cancer recurrence [222].

**Figure 4:**
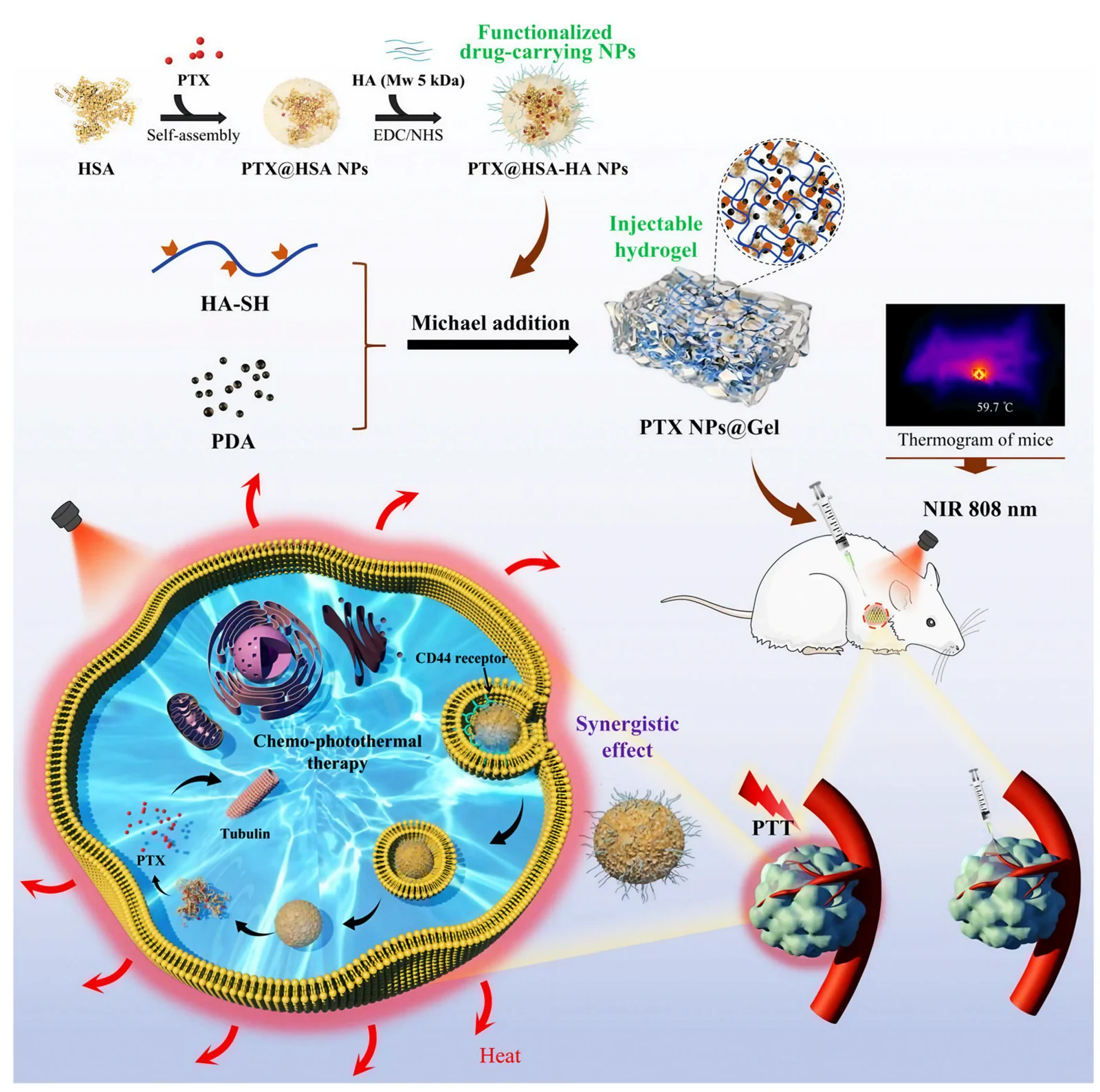
The PTX@HSA-HA hydrogel encapsulates PTX nanoparticles and PDA with excellent photothermal properties, targeting breast cancer to exert synergistic chemo-photothermal therapy [184].

In addition, coupling hyaluronic acid active ligand-modified nanoparticles with a hydrogel can raise the uptake rate by attaching to the CD44 receptor overexpressed on the membrane of tumor cells, thereby enhancing the targeting and efficiency of drug delivery. With the aim of improving nanoparticle uptake and overcoming the shortcomings of conventional injected nanoparticles, Xin Meng et al. innovatively employed HA-functionalized nanoparticles (HA-SH) and, through oxidizing the catechol groups of dopamine into the quinone form, performed a Michael addition with the thiol groups in HA-SH, prompting polydopamine (PDA) nanoparticles to bridge across the polymer chains and produce an injectable hydrogel possessing adhesive properties [184]. Here, the hydrogel encapsulates paclitaxel (PTX)-containing nanoparticles (PTX@HSA-HA) and simultaneously contains PDA, which has excellent photothermal properties, to further exert synergistic chemo-photothermal therapy. The results showed that, with the HA-functionalized nanoparticles and the hydrogel serving as carriers, the chemotherapeutic drug achieved precise targeting of tumor cells and a controlled drug-release effect. Meanwhile, under the action of PDA, after near-infrared irradiation, the hydrogel temperature rose above 58 °C, inducing tumor cell apoptosis. Therefore, this injectable hydrogel offers promise as a versatile therapeutic platform for the cooperative integration of photothermal and chemotherapeutic approaches (Figure 4).

Active modification of ligands improves targeting accuracy and, when combined with chemotherapeutic-photothermal therapy, also inhibits tumor growth. pH-sensitive hydrogels with active ligand modification can also improve tumor suppression efficiency by enabling controlled drug release in combination with chemotherapeutic-photothermal therapy. Jayeeta Bhaumik has developed a laser-sensitive photostructured hydrogel (DOX@CDs-ZnPc-PP H) that is capable of releasing both chemotherapeutic (DOX) and phototherapeutic (zinc phthalocyanine, ZnPc) drugs to achieve a synergistic effect against cancer [223]. This hydrogel responds to the acidic microenvironment of the tumor and triggers the release of both chemotherapeutic and phototherapeutic agents. The results show that pH-sensitive, laser-assisted drug release induces cytotoxicity in vitro through the formation of reactive oxygen species, enabling synergistic combination therapy of chemotherapy and photodynamic. Consequently, the new photostructured hydrogel system, which requires only a single treatment per cell, exhibits the highest inhibition rate due to synergistic effects and offers a promising concept for the effective treatment of cancer.

#### Comparison of Combination Modalities

Four further modalities are being combined with injectable depots. Each addresses a specific limitation of light-based therapy.

Chemodynamic therapy converts endogenous hydrogen peroxide into hydroxyl radicals by Fenton or Fenton-like catalysis with iron, copper, or manganese species. It requires neither light nor oxygen. Its rate depends on peroxide availability, on pH, and on GSH, which quenches the radicals produced. The most effective designs therefore deplete GSH as part of the catalytic cycle. The HA-TA/MXene@Cu system illustrates this clearly. GSH depletion reduces Cu^2+^ to the catalytically active form, radical production drives lipid peroxidation and ferroptosis, and MXene adds photothermal heating that accelerates the Fenton reaction [116]. Nanozyme-loaded depots follow the same logic. Hu and colleagues integrated a CuMnOx nanozyme, CuO_2_ nanoflowers, and the photosensitizer IR820 into an injectable thermosensitive hydrogel for synergistic breast cancer therapy and postsurgical adjuvant treatment [218]. Because catalytic metals benefit from being retained at the lesion, the depot format suits this modality particularly well.

Sonodynamic therapy uses focused ultrasound to activate a sonosensitizer. It offers the one advantage that photothermal and photodynamic therapy cannot, namely penetration to a depth of several centimeters. This matters for large or deep-seated breast tumors and for bone metastases. The hybrid membrane-coated sonosensitizer nanoparticles were developed for this indication 32. The Pt-TiO_2_ depot combines sonodynamic action with pH-responsive release and immune activation [224]. Like photodynamic therapy, sonodynamic therapy consumes oxygen and is attenuated in the hypoxic core. Oxygen-generating components are therefore often co-formulated.

Radiosensitization is the most clinically pragmatic combination. Adjuvant radiotherapy is already standard after breast-conserving surgery, and a depot placed in the tumor bed during resection requires no additional procedure. The MMP-responsive sunitinib nanoparticle hydrogel improved radiosensitivity and reduced local recurrence [111]. High-atomic-number nanoparticles, catalytic metals, and hypoxia-relieving agents serve the same purpose. Wang and colleagues prepared a copper-doped polypyrrole hydrogel with tumor catalytic activity for combined thermo-radiotherapy in the second near-infrared window [217]. One design constraint is specific to this combination. A depot must maintain the sensitizing concentration across several weeks of fractions, which is considerably longer than the release window of most reported systems.

Gene therapy completes the set. The RNA triple-helix and aptamer-crosslinked systems silence targets that small molecules cannot address [179]. Delivering silencing and chemotherapy from a single depot allows the resistance pathway and the cytotoxic insult to be addressed at the same time, as in the siCALD1 and anthracycline construct [164].

A critical observation applies to all of these combinations. Synergy is claimed in almost every study cited in this section, but it is rarely demonstrated formally. Combination index, Bliss independence analysis, or full factorial comparison at matched doses are seldom reported. The chemo-photothermal data quoted above are unusual in providing single-modality controls. More often the combination is compared only with free drug and with untreated animals, so that an additive effect cannot be distinguished from a synergistic one. The modalities also differ in ways that should guide their pairing. Near-infrared photothermal and photodynamic therapy reach only about one centimeter of tissue and require repeated irradiation. Sonodynamic therapy penetrates further but depends on oxygen. Chemodynamic therapy is oxygen-independent but is limited by the endogenous peroxide supply. Radiotherapy is unrestricted in depth but is constrained by the cumulative dose to normal tissue. A rational combination therefore pairs modalities whose limitations are complementary rather than overlapping, and states which limitation each partner is intended to relieve.

Based on the above, other types of multimodal combination therapy also exert synergistic therapeutic effects in breast cancer to enhance tumor inhibition. Meanwhile, the hydrogel design featured in numerous multimodal combination therapies parallels that used in the chemo-photothermal therapy combination—for instance, by reacting to several tumor microenvironmental traits or by introducing ligand modification into the hydrogel—so as to additionally improve the accurate targeted conveyance of drugs to tumor tissue and elevate local drug utilization. The applications of various multimodal therapy combinations in breast cancer treatment are compiled below in Table 8.

## 9. Current Limitations, Biosafety and Translational Challenges

The strategies described above are almost all at the preclinical stage. Before any of them reaches a patient, a set of problems has to be solved that has little to do with responsiveness or targeting. These problems are addressed here in three groups: manufacture, biosafety, and regulation.

### 9.1. Manufacturing, Reproducibility, Sterilization and Storage

Scale-up is the first obstacle. Most published depots are prepared at the milligram scale by manual mixing of two precursor solutions. Transferring such a procedure to pharmaceutical manufacture requires control of mixing rate, temperature, and gelation time, all of which alter the final network. Nanocomposite systems are harder still, because the nanocarrier must first be produced to a defined size and loading, and then dispersed without aggregation.

Batch-to-batch reproducibility follows directly from the choice of material. Natural polymers vary in molecular weight, degree of substitution, and endotoxin content between lots, and these differences change the modulus and the degradation rate of the resulting gel [231]. Synthetic polymers are more consistent but are not free of variability, since the block length distribution of a triblock copolymer determines the sol–gel transition temperature [232]. Variability of this kind is a recognized barrier for cell-laden and nanoparticle-laden systems in particular.

Sterilization is frequently omitted from preclinical reports, yet no method is neutral. Steam autoclaving hydrolyses polyesters and denatures proteins. Gamma irradiation causes chain scission or additional crosslinking and therefore shifts the modulus and the degradation profile. Ethylene oxide leaves residues that must be removed and validated. Sterile filtration of the precursor solution through a 0.22 micrometer membrane is the mildest option, but it is possible only when the precursor is of low viscosity and free of particles, which excludes most nanocomposite formulations [233]. Aseptic processing is then the remaining route, and it raises cost considerably.

Storage stability is a related problem. Thermosensitive precursors must be kept refrigerated in the sol state, which imposes a cold chain. Dynamic covalent networks continue to exchange bonds during storage, so their rheology drifts. Protein, nucleic acid, and cell components have their own shelf-life constraints. Lyophilization with reconstitution at the bedside is the usual solution, but it must be shown not to alter the network after rehydration [234].

### 9.2. Biosafety: Toxicity, Immunogenicity and Degradation Products

Local tolerance is generally good for the polymers used here, but the components added to make a depot functional deserve closer scrutiny than they usually receive. Cationic polymers such as chitosan and polyethyleneimine disrupt cell membranes at high charge density, and the same property that condenses nucleic acids also causes cytotoxicity. Unreacted electrophiles left after Michael addition, residual photoinitiator, and residual crosslinker are all potential irritants and should be quantified rather than assumed to be consumed [235].

Degradation products matter as much as the parent material. Polyester blocks in PLGA-PEG-PLGA release lactic and glycolic acid, which lowers the local pH and may itself trigger acid-labile bonds elsewhere in the network. Aldehyde-bearing polysaccharides used for Schiff base crosslinking can release free aldehyde as the network erodes. Inorganic components present the least resolved problem, since gold, bismuth, MXene, and iron oxide particles are cleared slowly, and their long-term fate after the organic network has disappeared is rarely followed beyond a few weeks [236].

Immunogenicity arises from several sources. Bacterial and plant-derived polysaccharides may carry endotoxin. Protein components such as horseradish peroxidase, bacterial Cas9, and antibody fragments can provoke an antibody response on repeat administration. Cell-membrane coatings derived from tumor cells raise a further question about residual nucleic acid. A depot intended to remain in place for weeks also creates a persistent inflammatory stimulus, and an excessively stiff gel promotes fibrous encapsulation, which both isolates the depot and impedes immune cell traffic [237].

Long-term biocompatibility is therefore assessed on a longer timescale than most studies use. Reporting histology of the implantation site, serum chemistry, and organ accumulation at the point when the network has fully degraded, rather than at the end of the efficacy experiment, would be a reasonable minimum.

### 9.3. Regulatory Barriers, Clinical Translation and Ways Forward

Regulatory classification is a practical difficulty. A drug-loaded hydrogel is a combination product, so the polymer and the drug are assessed together, and the biomaterial must satisfy device requirements while the drug satisfies pharmaceutical ones. Cell-laden depots add a third layer. Approval therefore requires a defined and reproducible manufacturing process, validated sterilization, established shelf life, and biocompatibility testing of the specific formulation, none of which can be inferred from published preclinical data. Placement is a further issue, since accurate delivery into an intact deep-seated lesion requires image guidance, whereas filling a resection cavity does not.

Several routes forward follow from these constraints. Simplifying formulation is the most direct. A two-syringe system built from a small number of pharmaceutically acceptable components is far easier to manufacture and sterilize than a multicomponent nanocomposite, and the polymer-nanoparticle depot used for cell delivery shows that simple systems can still perform well [238]. Using excipients that already have regulatory precedent, such as poloxamer, hyaluronic acid, alginate, and gelatin, reduces the toxicological burden. Replacing components of uncertain fate with degradable or endogenous alternatives is also useful, and the substitution of iron for gadolinium in MRI-visible gels is an example [218]. Standardized reporting of network parameters, loading, residence time, and degradation would allow candidates to be selected on comparable evidence. Finally, the postoperative tumor bed is the most realistic first indication, because the cavity is accessible during surgery, the depot can be placed without additional intervention, and the clinical endpoint of local recurrence is well defined.

## 10. Conclusions and Future Perspectives

Local recurrence, systemic toxicity, tumor heterogeneity, drug resistance, and the immunosuppressive microenvironment have long constrained the efficacy of conventional surgery, chemotherapy, and radiotherapy in breast cancer. In recent years, leveraging the injectability, in situ gelation, and good biocompatibility of hydrogels, researchers have developed a variety of controlled-release systems for the local and precise delivery of drugs to breast cancer. Compared with systemic administration, injectable hydrogels form a local drug depot at the tumor site through minimally invasive administration, prolonging drug retention and reducing systemic toxicity. Based on the pathobiochemical features of the breast cancer tumor microenvironment—weak acidity (pH 6.5–6.9), elevated GSH, overexpression of hyaluronidase and matrix metalloproteinases, oxidative stress, hypoxia, and elevated temperature—researchers have introduced pH-sensitive groups, disulfide bonds, thioketal and boronate ester linkages, enzyme-cleavable peptides, and thermosensitive segments into the hydrogel network to achieve on-demand release of drugs at the tumor site; targeting highly expressed receptors such as CD44, the folate receptor, integrins, EGFR, the transferrin receptor, and HER2, they have modified hydrogels with small molecules, polysaccharides, proteins/peptides, aptamers, and biomimetic cell membranes, upgrading the hydrogel from passive retention to active recognition through receptor-mediated endocytosis. On this basis, hydrogels further serve as local reservoirs for cancer vaccines, immune checkpoint inhibitors, and CAR-T/CAR-NK cells, transforming local intervention into systemic antitumor immunity, and, combined with biosensing technologies, imaging contrast agents, and multimodal combination therapy, demonstrate potential for integrated diagnosis and therapy. It can thus be seen that injectable hydrogels are not merely a drug container, but an engineerable therapeutic platform onto which multiple functions can be superimposed; among these, dual-functionalized hydrogels that combine both stimulus responsiveness and active targeting, with their dual advantages in sustained release and targeted delivery, represent the most translationally promising research direction at present.

Despite these abundant achievements, injectable hydrogels still face several limitations before clinical translation: the intratumoral and inter-patient heterogeneity of tumor microenvironment signals renders the drug-release behavior of single-responsive hydrogels difficult to predict and insufficiently reproducible between batches; targets such as CD44, EpCAM, and the folate receptor are also expressed on normal cells, posing a risk of off-target effects, while protein/peptide and nucleic acid payloads face challenges in stability and immunogenicity; the dense stroma and hypoxia of solid tumors limit the infiltration and survival of CAR-T/CAR-NK cells; in addition, the long-term biocompatibility of hydrogels, the metabolism of their degradation products, and the scale-up production and quality control of complex systems still await systematic evaluation. In addition, a methodological limitation cuts across the entire field: network parameters and rheological data are reported too rarely for published systems to be compared with one another, responsive designs are seldom benchmarked against non-responsive depots of matched modulus and loading, and claims of synergy are usually made without formal combination analysis. Progress toward translation depends at least as much on tightening these conventions as on introducing new chemistries. To address these issues, future work should develop multi-responsive and logic-gated designs as well as dual-target or in situ activatable ligand strategies, optimize the mechanical and degradation behavior of the gel to maintain immune cell viability, and strengthen validation in organoids, patient-derived xenografts (PDX), and humanized models, establishing standardized quality-control workflows and incorporating chemistry, manufacturing, and controls and regulatory considerations at an early stage.

Several developments are likely to reshape the way in which such systems are designed. The formulation space of an injectable depot is large. Polymer identity, degree of substitution, crosslinker ratio, and nanoparticle loading all interact, and their combined effect on modulus, degradation, and release cannot be explored efficiently one factor at a time. Machine learning models trained on rheological and release data offer a means of predicting these properties from composition, and of identifying the compositions that satisfy a required retention time and release profile. Digital manufacturing and three-dimensional bioprinting allow depots to be fabricated with a defined internal architecture and a spatially graded composition, so that stiffness, porosity, and release rate can be varied within a single construct. Printed tumor models that incorporate stromal and immune components also provide a more discriminating test bed than monolayer culture. For a depot placed in a resection cavity of individual geometry, fabrication from preoperative imaging is a natural extension, although sterility, timing, and regulatory requirements remain demanding. Finally, breast cancer is not a single disease. Matching depot design to molecular subtype, and ultimately to the receptor profile and microenvironment of an individual lesion, appears a more realistic route to precision than the pursuit of a single universally responsive material.

Overall, with the deepening intersection of materials chemistry, tumor immunology, computational design, and clinical engineering, hydrogels will evolve along the directions of precision, intelligence, and integration, and injectable hydrogels that are mechanistically clear, safe, controllable, and readily scalable are poised to become an important pillar of local precision therapy and integrated theranostics for breast cancer, ultimately improving patient prognosis.

## Figures and Tables

**Table 1:** Classification of injectable hydrogels used for breast cancer delivery and their principal physicochemical characteristics.

Category	Representative Materials	Network Formation	Typical Physicochemical Profile	Principal Limitations
Natural polymer	Hyaluronic acid, chitosan, alginate, gelatin, collagen, dextran	Ionic crosslinking, Schiff base, enzymatic crosslinking	Highly hydrated, storage modulus commonly reported in the 10^1^–10^3^ Pa range; enzymatically degradable; intrinsic bioactivity (e.g., HA–CD44)	Batch-to-batch variability; limited mechanical strength; degradation rate difficult to decouple from stiffness [38,39]
Synthetic polymer	Poloxamer 407, PNIPAM, PLGA-PEG-PLGA, PEG, PVA	Thermally driven micellar packing, photo-crosslinking, click chemistry	Reproducible composition; tunable sol–gel transition near 37 °C; modulus and erosion time adjustable over an order of magnitude	Little intrinsic bioactivity; acidic degradation products for polyester blocks; often requires functionalisation [40,41]
Hybrid	Chitosan–PEG, PVA–MOF, alginate–PNIPAM, polysaccharide–peptide conjugates	Dual physical/chemical crosslinking	Combines loading capacity of the inorganic or synthetic phase with the tissue affinity of the natural phase; markedly higher drug payload	Increased formulation complexity; interfacial stability and reproducibility harder to control [42]
Supramolecular	Ultrashort and lipidated peptides, β-cyclodextrin–adamantane, DNA/aptamer assemblies	Non-covalent self-assembly, host–guest inclusion	Gelation under physiological conditions; intrinsically shear-thinning and self-healing; molecularly defined	Low fracture strength; concentration-dependent stability; susceptible to dilution and proteolysis [43,44,45]
Nanocomposite	Liposome-, polymeric nanoparticle-, MOF-, MXene-, Au-, Fe_3_O_4_-, or vesicle-loaded gels	Embedding of nanocarriers within any of the above networks	Two-stage release (gel → nanocarrier → cell); adds photothermal, magnetothermal, catalytic or imaging function; suppresses burst release of hydrophobic drugs	Nanoparticle migration and aggregation; long-term fate of inorganic components unresolved [46,47]
Self-healing/dynamic covalent	Imine, acylhydrazone, boronate ester, disulfide, metal–catechol networks	Reversible covalent or coordination bonds	Recovers network integrity after needle shear; conforms to irregular resection cavities; bond lability confers pH/ROS/redox responsiveness	Continuous bond exchange causes creep and gradual erosion; responsiveness and mechanical stability are coupled and cannot be tuned independently [48,49]

**Table 2:** In situ gelation mechanisms available for injectable breast cancer depots.

Mechanism	Chemistry/Driving Force	Typical Gelation Trigger and Rate	Cargo Compatibility	Main Constraints
Thermosensitive	Hydrophobic association above LCST (poloxamer, PNIPAM, PLGA-PEG-PLGA)	Body temperature; seconds to a few minutes	Excellent, including proteins and cells	Sol state must be kept cold before injection; erosion-dominated, relatively short residence [60,61,62]
Ionic crosslinking	Ca^2+^–alginate egg-box; chitosan–β-glycerophosphate	Ion exchange on contact with tissue fluid; seconds to minutes	Excellent	Progressive ion exchange in vivo leads to gradual softening [63,64]
Host–guest	β-cyclodextrin–adamantane or –azobenzene inclusion	Mixing; instantaneous, reversible	Excellent; shear-thinning protects cells	Low modulus; dilution-sensitive [65,66]
Supramolecular self-assembly	Peptide β-sheet stacking, π–π and hydrogen bonding, nucleobase pairing	Salt or pH change on injection; seconds to minutes	Excellent	Limited fracture strength; proteolytic susceptibility [67,68,69]
Schiff base	Aldehyde + amine → imine	Mixing at neutral pH; minutes	Good, but consumes free amines of proteins	Imine hydrolysis in acid couples persistence to tumor pH [70,71]
Michael addition/thiol–ene	Thiol + acrylate, maleimide or vinyl	Mixing at pH 7.4; seconds to minutes	Good, with the caveat of cysteine modification	Residual unreacted electrophiles must be controlled [72,73]
Click (SPAAC, tetrazine–TCO)	Strain-promoted or inverse electron-demand cycloaddition	Mixing; seconds to minutes, catalyst-free	Excellent, bio-orthogonal	Cost and synthetic burden of functionalized precursors [74,75]
Photo-crosslinking	Radical polymerization of methacrylated GelMA/HAMA	UV or visible light; seconds under illumination	Poor for nucleic acids and cells	Requires light access and photoinitiator; radical damage [76,77]
Enzymatic	HRP/H_2_O_2_–tyramine coupling; transglutaminase	Enzyme mixing at 37 °C; minutes	Excellent	Exogenous enzyme and, for HRP, H_2_O_2_ must be cleared [78,79]

**Table 5:** Molecular targets and ligand types used for the active targeting of breast cancer, and representative systems.

Target	Ligand Type	Basis of Expression in Breast Cancer	Representative System	Main Limitation	Refs.
CD44	Hyaluronic acid (polysaccharide)	Activated and strongly expressed on tumor cells and cancer stem cells, dormant on healthy cells	HA-ATRA-AuNP composite hydrogel that depletes cancer stem cells and combines with PTT	Also expressed on normal epithelium and hematopoietic cells	[23,24,149,150]
Folate receptor	Folic acid (small molecule)	Elevated in rapidly dividing cells to meet nucleotide demand; low in normal tissue	Folic acid/PEG-glycidyl methacrylate chitosan hydrogel delivering paclitaxel; GOFA-DOX/HACPN thermosensitive hydrogel	Present in kidney and lung; weakly expressed in much of triple-negative disease	[25,147,152,153]
HER2	Aptamer, antibody, affibody	Amplified or overexpressed in about 20–25% of breast cancers	Dual-aptamer DNA hydrogel (DTA-H) co-delivering DOX and aptamer drug; affibody-DNA gold nanoparticles	Applicable only to the HER2-positive subtype	[27,28,166,167]
Integrins (alpha-v-beta-3, alpha-5-beta-1)	RGD peptide and its cyclic and tumor-penetrating derivatives	Upregulated on tumor neovasculature and invasive tumor cells	Cyclic RGD-conjugated solid lipid nanoparticles; RGD motifs used as adhesion sites in cell-laden gels	Cyclic RGD prolongs retention but increases mononuclear phagocyte uptake, lowering total tumor accumulation	[168,169]
EGFR (HER1)	GE11 peptide, cetuximab fragments, affibody	Overexpressed in a substantial fraction of triple-negative disease	GE11 self-assembled nanoplatform loaded with DOX; HER1-CAR-T cells combined with an H_2_O_2_-responsive hydrogel	Expressed in skin and gastrointestinal epithelium; heterogeneous within tumors	[170,171,172]
Transferrin receptor	Transferrin, mimetic peptides	Upregulated to meet the iron demand of proliferating cells	Transferrin-targeted polymeric nanoparticles for phototriggered treatment	Broadly expressed, including at the blood–brain barrier and in erythroid precursors	[22,29]
EpCAM	Aptamer	Expressed on tumor cells of epithelial lineage	Dual-aptamer (EpCAM and CD63) hydrogel for isolation and detection of tumor-derived exosomes	Expressed on healthy epithelium; heterogeneous across tumor cells	[173,174]
CCR9	CCL25 chemokine (protein ligand)	Present on CD8^+^ T cells rather than on tumor cells	Thermosensitive hydrogel releasing CCL25 to recruit CD8^+^ T cells into TNBC	Acts on immune cells, so the antitumor effect is indirect	[156,157]
CD276 and other tumor antigens	Single-chain variable fragment (scFv)	Elevated on senescence-escaping cancer stem cells in anthracycline-resistant disease	Anti-CD276 scFv-engineered biomimetic nanovesicles delivered from a thermosensitive hydrogel	Cost, immunogenicity, and partial loss of activity on conjugation	[164]
Homotypic and hybrid membranes (ligand-free)	Cell membrane coating	Uses the native surface proteome of erythrocytes, platelets, leukocytes, or tumor cells	Bacterial outer membrane vesicle-cancer cell hybrid membrane coating sonosensitizer nanoparticles for bone metastasis	Difficult to characterize and standardize; regulatory concerns for cancer-cell-derived material	32
Dual targeting	Two ligands on one carrier	Addresses intratumoral heterogeneity of receptor expression	Anti-CD44 and EGFR dual-targeted solid lipid nanoparticles delivering doxorubicin	Increased synthetic complexity; risk of reduced ligand density for each target	[175]

**Table 6:** Principal therapeutic agents used in breast cancer, the limitations of conventional administration, and the advantages offered by hydrogel-based local delivery.

Agent (Class)	Typical Dose in Conventional Use	Principal Limitations of Conventional Administration	Advantage Offered by Hydrogel Delivery	Example in This Review
Doxorubicin (anthracycline)	60 mg/m^2^ intravenously every 3 weeks; cumulative dose limited to about 450–550 mg/m^2^	Dose-limiting cardiotoxicity, myelosuppression, alopecia; poor tumor selectivity	Local depot lowers the plasma peak that drives cardiotoxicity and sustains intratumoral exposure; also induces immunogenic cell death in situ	Chemo-photothermal and chemo-immunotherapy depots [182,183,184]
Epirubicin (anthracycline)	90–100 mg/m^2^ intravenously every 3 weeks	Short half-life and pronounced systemic toxicity	Prodrug nanoassembly releases the drug on encountering intracellular GSH, improving the therapeutic window	Disulfide-linked lipophilic prodrug nanoassemblies [118]
Paclitaxel (taxane)	175 mg/m^2^ every 3 weeks or 80 mg/m^2^ weekly, intravenously	Very poor aqueous solubility, requiring Cremophor-based vehicles that cause hypersensitivity; peripheral neuropathy	Nanocrystal or albumin-bound forms can be dispersed in the gel without a solubilizing vehicle, and release is spread over weeks	Paclitaxel nanocrystal thermogel and PTX@HSA-HA depot [134,184]
Docetaxel (taxane)	75–100 mg/m^2^ intravenously every 3 weeks	Febrile neutropenia, fluid retention, hypersensitivity	Co-loading with a second agent in one depot allows a lower absolute dose	Dual-drug magnetic hydrogel [141]
5-Fluorouracil (antimetabolite)	500–600 mg/m^2^ intravenously, repeated within the cycle	Very short plasma half-life, mucositis, myelosuppression	High loading in a porous hybrid network and pH-triggered release maintain the local concentration	CuBTC-PVA hybrid hydrogel, loading raised from 80 to 261.4 mM [100]
Sunitinib (multikinase inhibitor)	37.5–50 mg daily, oral	Hypertension, hand-foot syndrome, fatigue on continuous dosing	Enzyme-triggered release confines antiangiogenic and radiosensitizing activity to the tumor bed	MMP-responsive nanoparticle hydrogel [111]
Raloxifene (selective estrogen receptor modulator)	60 mg daily, oral	Low solubility, extensive first-pass metabolism, poor tumor targeting	Acid-triggered swelling and charge reversal raise local bioavailability	Chitosan-glyceryl monooleate invasome hydrogel, intratumoral bioavailability increased 4.07-fold [98]
Abemaciclib (CDK4/6 inhibitor)	150 mg twice daily, oral	Diarrhea, neutropenia; continuous dosing required	Sustained local release induces immunogenic cell death and supports neoadjuvant immunotherapy	Supramolecular peptide-drug hydrogel with NLG919 [159]
Anti-PD-L1 and anti-PD-1 antibodies	Atezolizumab 840 mg every 2 weeks or pembrolizumab 200 mg every 3 weeks, intravenously	Immune-related adverse events in a substantial minority of patients; limited intratumoral concentration	Erosion-controlled local release maintains blockade within the lesion at a far smaller absolute dose	aPD-L1 co-delivered with DOX from a silk-elastin-like protein gel [182]
Metformin (repurposed agent)	500–2000 mg daily, oral	Low potency at the tumor, gastrointestinal intolerance, wide systemic distribution	Imine conjugation to the network gives acid-triggered release at the resection site	CMCS/OHA-Met hydrogel for recurrence prevention [101]

**Table 7:** Payload classes delivered from injectable hydrogels and the design constraints they impose.

Payload Class	Dominant Constraint	Compatible Gelation Chemistry	Retention Strategy	Outstanding Issue	Refs.
Small-molecule chemotherapeutics	Burst release of hydrophilic agents; poor loading of hydrophobic agents	All mechanisms in Table 2	Electrostatic binding; nanocrystal, micelle or liposome pre-formulation	Local concentration-time profiles are rarely measured	[63,67,128]
Natural products	Poor aqueous solubility; modest potency	All mechanisms in Table 2	Nanoparticle or cyclodextrin inclusion; prodrug conjugation	Weak dose–response data and few head-to-head comparisons with standard cytotoxics	[42,44,48,55,65,137]
Proteins and peptides	Denaturation and covalent modification during crosslinking	Thermal, ionic, host-guest, self-assembly	Affinity or heparin-mimetic anchoring; electrostatic complexation	Activity after release is seldom verified	[86,88,102]
Nucleic acids (siRNA, mRNA, DNA)	Nuclease degradation; no unassisted cellular entry	Mild physical chemistries; DNA self-assembly	Polycation or lipid condensation; covalent cholesterol anchoring	Endosomal escape and durability of silencing	[91,95,96]
Antibodies and antibody-drug conjugates	Aggregation at interfaces; limited injectable dose	Thermal, ionic, supramolecular	Erosion-controlled release from a dense network	Achievable local dose relative to the systemic standard of care	[107,109]
Exosomes and extracellular vesicles	Integrity of the membrane and surface proteome	Non-covalent networks only	Hydrogen bonding and electrostatic immobilization	Standardization, characterization, and scale-up	[80,93]
Gene-editing complexes (Cas9 RNP)	Size, charge, and immunogenicity of the bacterial protein	Mild physical chemistries; cationic networks	Cationic or lipid complexation; charge-matched network	Almost unexplored in breast cancer; in vivo editing efficiency unproven	[163,164]
Living cells (CAR-T, CAR-NK)	Viability during injection; proliferation and egress	Shear-thinning, yield-stress supramolecular networks	Adhesion motifs, cytokine co-delivery, porosity permitting egress	Persistence, manufacturing, and regulatory complexity	[115,118,145]

**Table 8:** Application of Multimodal Combination Therapy in Breast Cancer.

Multimodal Therapy Type	TME-Responsive Elements	Active Targeting Ligands	Hydrogel Matrix	Nanocarrier	Therapeutic Agent(s)	Primary Function
Chemotherapy-Photothermal Therapy	/	/	Chitosan-Catechol Hydrogel (CAT-Gel)	Chitosan (CS)	Doxorubicin Hydrochloride (DOX) and Docetaxel (DTX)	Treatment of breast cancer [225].
Chemotherapy-Photothermal Therapy	PH	/	Prussian blue modified N-carboxyethyl chitosan (PBCEC) and oxidized sodium alginate (PB/DOX hydrogel)	/	DOX	Preventing breast cancer metastasis and recurrence and treating wound infections [226].
Chemotherapy-Photothermal Therapy	GSH	/	L/M-containing injectable composite hydrogel	liquid metal (LM)	6-mercaptopurine (MP)	Inhibit tumor growth and treat breast cancer [227].
Photothermal-Photodynamic therapy	PH	/	Sodium alginate oxidized (OSA) and hydroxypropyl chitosan (HPCS)	BSA-MoS	/	Inhibit tumor growth and treat breast cancer [228].
Chemotherapy-Photothermal Therapy-Photodynamic Therapy	/	PC10A	PC10A/DOX/MoS2	MOS	DOX	Hydrogels with PC10A/DOX/MoS2 and laser irradiation can activate anti-tumor immune responses and inhibit the growth of primary 4T1 breast tumors [229].
Chemotherapy-Photothermal Therapy-Immunotherapy	Temperature	/	PCur/IND@Gel	Dopamine (PDA)-Curcumin (Cur) nanoparticles (PCur-NPs)	Cur, indoximod, IND	Induced significant apoptosis in 4T1 tumors, enhanced CD8^+^ and CD4^+^ anti-tumor immune responses, significantly inhibited tumor growth, and increased the survival rate of 4T1 tumor-bearing mice [230].
Chemodynamic-Photothermal Therapy	GSH	HA (CD44)	Hyaluronic acid–tannic acid hydrogel	MXene@Cu nanosheets	Cu-based Fenton catalyst	GSH depletion drives Cu^2+^ reduction and hydroxyl radical generation, inducing ferroptosis; MXene provides photothermal amplification [116].
Sonodynamic-Immunotherapy with fluorescence imaging	pH (boronate ester)	/	Boronate ester crosslinked nanocomposite hydrogel	Pt–TiO_2_ nanoparticles	Sonosensitizer and fluorescent probe	Ultrasound-triggered ROS generation with acid-triggered release and in situ fluorescence reporting in cold breast tumors [224].
Photothermal-Immunotherapy with MRI contrast	/	/	Plug-and-play injectable hydrogel	Prussian blue–manganese	Mn^2+^	Photothermal ablation combined with Mn^2+^-mediated immune activation and magnetic resonance visualization of the depot [149].
Radiosensitization-Antiangiogenic therapy	MMP	/	MMP-responsive peptide-crosslinked hydrogel	Sunitinib nanoparticles	Sunitinib	Enzyme-triggered release of an antiangiogenic agent enhances radiosensitivity and prevents local recurrence after resection; catalytic copper-doped depots serve the same purpose in combined thermo-radiotherapy [217].
Chemodynamic-Photothermal-Chemotherapy	pH and H_2_O_2_	/	Injectable thermosensitive nanozyme hydrogel	CuMnOx nanozyme and CuO_2_ nanoflowers	IR820	Microenvironment-responsive nanozyme depot combining catalytic radical generation with photothermal action for breast cancer therapy and postsurgical adjuvant treatment [218].

## Data Availability

No new data were created or analyzed in this study. Data sharing is not applicable to this article.

## Abbreviations

The following abbreviations are used in this manuscript:

TME	tumor microenvironment
GSH	glutathione
ROS	reactive oxygen species
ECM	extracellular matrix
MMP	matrix metalloproteinase
HAase	hyaluronidase
HA	hyaluronic acid
FR	folate receptor
HER2	human epidermal growth factor receptor 2
TNBC	triple-negative breast cancer
DOX	doxorubicin
PTX	paclitaxel
PTT	photothermal therapy
PDT	photodynamic therapy
ICI	immune checkpoint inhibitor
CAR-T	chimeric antigen receptor T cell
CAR-NK	chimeric antigen receptor natural killer cell
TAM	tumor-associated macrophage
PDX	patient-derived xenograft
CMC	chemistry, manufacturing, and controls
